# Qualification of a multiplexed tissue imaging assay and detection of novel patterns of HER2 heterogeneity in breast cancer

**DOI:** 10.1038/s41523-023-00605-3

**Published:** 2024-01-02

**Authors:** Jennifer L. Guerriero, Jia-Ren Lin, Ricardo G. Pastorello, Ziming Du, Yu-An Chen, Madeline G. Townsend, Kenichi Shimada, Melissa E. Hughes, Siyang Ren, Nabihah Tayob, Kelly Zheng, Shaolin Mei, Alyssa Patterson, Krishan L. Taneja, Otto Metzger, Sara M. Tolaney, Nancy U. Lin, Deborah A. Dillon, Stuart J. Schnitt, Peter K. Sorger, Elizabeth A. Mittendorf, Sandro Santagata

**Affiliations:** 1 Division of Breast Surgery, Department of Surgery, Brigham and Women’s Hospital, Boston, MA 02115 USA; 2 Breast Tumor Immunology Laboratory, Dana-Farber Cancer Institute, Boston, MA 02215 USA; 3 Ludwig Center for Cancer Research at Harvard, Harvard Medical School, Boston, MA 02215 USA; 4 Laboratory of Systems Pharmacology, Department of Systems Biology, Harvard Medical School, Boston, MA 02215 USA; 5 Department of Pathology, Hospital Sírio Libanês, São Paulo, SP 01308-050 Brazil; 6 Department of Pathology, Brigham and Women’s Hospital, Harvard Medical School, Boston, MA 02115 USA; 7 Department of Molecular Diagnostics, Sun Yat-sen University Cancer Center, Guangzhou, China; 8 Breast Oncology Program, Dana-Farber/Brigham and Women’s Cancer Center, Boston, MA 02215 USA; 9 Department of Data Science, Dana-Farber Cancer Institute, Boston, MA 02215 USA

**Keywords:** Cancer imaging, Tumour heterogeneity

## Abstract

Emerging data suggests that HER2 intratumoral heterogeneity (ITH) is associated with therapy resistance, highlighting the need for new strategies to assess HER2 ITH. A promising approach is leveraging multiplexed tissue analysis techniques such as cyclic immunofluorescence (CyCIF), which enable visualization and quantification of 10–60 antigens at single-cell resolution from individual tissue sections. In this study, we qualified a breast cancer-specific antibody panel, including HER2, ER, and PR, for multiplexed tissue imaging. We then compared the performance of these antibodies against established clinical standards using pixel-, cell- and tissue-level analyses, utilizing 866 tissue cores (representing 294 patients). To ensure reliability, the CyCIF antibodies were qualified against HER2 immunohistochemistry (IHC) and fluorescence in situ hybridization (FISH) data from the same samples. Our findings demonstrate the successful qualification of a breast cancer antibody panel for CyCIF, showing high concordance with established clinical antibodies. Subsequently, we employed the qualified antibodies, along with antibodies for CD45, CD68, PD-L1, p53, Ki67, pRB, and AR, to characterize 567 HER2+ invasive breast cancer samples from 189 patients. Through single-cell analysis, we identified four distinct cell clusters within HER2+ breast cancer exhibiting heterogeneous HER2 expression. Furthermore, these clusters displayed variations in ER, PR, p53, AR, and PD-L1 expression. To quantify the extent of heterogeneity, we calculated heterogeneity scores based on the diversity among these clusters. Our analysis revealed expression patterns that are relevant to breast cancer biology, with correlations to HER2 ITH and potential relevance to clinical outcomes.

## Introduction

Over the past decade, there has been an increasing awareness of the key roles played by intratumor heterogeneity (ITH) and the tumor microenvironment (TME) in breast cancer^1–3^. Thus, there is a pressing need to gain a better understanding of the role played by molecular variation in the development and progression of tumors. Recently developed technologies that permit the detailed characterization of complex spatial relationships among tumor, immune, and stromal cells at single-cell resolution hold substantial potential for providing critical insight into the TME, which may help identify opportunities to improve clinical care. Multiplexed tissue imaging methods address these needs by building upon the extensive experience gained over many years by pathologists using immunohistochemistry (IHC). The routine assessment of estrogen receptor (ER) and progesterone receptor (PR) levels using IHC has established them as critical prognostic markers and strong predictors of response to endocrine therapy^4,5^. Similarly, human epidermal growth factor receptor 2 (HER2) expression helps identify patients who are more likely to respond to anti-HER2-targeted therapy. IHC is commonly used for HER2 protein expression analysis, and fluorescence in situ hybridization (FISH) serves as a complementary approach to confirm HER2 gene amplification. The detection of ER, PR, and HER2 by IHC has been instrumental in determining appropriate therapeutic approaches for breast cancer patients. However, more advanced quantification methods and single-cell analysis have the potential to further refine and personalize treatment strategies.

Previous studies have extensively documented the ITH of ER, PR, and HER2 expression using IHC^6^. However, a comprehensive characterization of the expression of these markers at the single-cell level has not yet been performed. In standard pathology practice, ER and PR IHC are scored at the whole tissue level, and the percentage of immunoreactive tumor nuclei is reported using a semiquantitative scoring system which categorizes samples as positive (≥10% of nuclei immunoreactive), low positive (≥1% to <10% of nuclei immunoreactive) or negative (<1% nuclei immunoreactive). Assessing HER2 expression involves a more complex scoring process that considers the intensity of immunoreactivity, the extent of membranous signal (partial or complete), and the proportion of positive cells. Along with semiquantitative scoring of HER2 expression (0, 1+, 2+, or 3+), HER2 FISH is utilized in most institutions to analyze equivocal samples (scored as 2+) following the guidelines set by the American Society of Clinical Oncology (ASCO)/College of American Pathologists (CAP)^7^. HER2 amplification, determined by FISH, is defined as a HER2/CEP17 ratio greater than 2.0 with an average HER2 copy number greater than 4.0 using a dual probe system or an average HER2 copy number of greater than 6.0 HER2 signals/cell using a single probe system. Around 15–20% of breast cancer cases are identified as HER2+ based on protein overexpression and/or gene amplification. Prior to the development of HER2-targeted therapies, HER2 positivity was associated with a poor prognosis^8–10^. Now, HER2 protein overexpression is the primary predictor of responsiveness to HER2-targeted therapies in breast cancer. However, despite the careful patient selection using IHC/FISH and the availability of contemporary HER2-targeted therapies, pathological complete response (pCR) is only observed in 30–56% of HER2+ patients receiving preoperative therapy^11–16^. Moreover, primary and acquired clinical resistance to these therapies has been increasingly reported^17^. Differences in pCR rates are partly associated with the hormone receptor (HR) status, where patients with HR+/HER2+ tumors are less likely to experience pCR compared to HR−/HER2+ tumors^16,18^. Importantly, even in tumors designated as HER2 3+ by IHC, not all cancer cells show high-level HER2 expression^19,20^, suggesting that HER2 heterogeneity may provide insights into therapeutic response.

HER2 ITH has been well documented in breast cancer^21^. HER2 overexpression and amplification can present a heterogeneous pattern, including HER2-positive and HER2-negative tumor cell subpopulations occurring within the same tumor^20,22^. Distinct patterns of cells with heterogenous HER2 status include “clustered” type, featuring the presence of two topographically distinct tumor clones of tumor cells, one harboring HER2 amplification and the other with normal HER2 status; “mosaic” type, displaying either diffuse intermingling of cells with different HER2 statuses; and “scattered type”, with isolated HER2-amplified cells in a HER2-negative tumor cell population^23–25^. According to the 2009 ASCO/CAP guidelines, HER2 genetic heterogeneity is defined as the presence of ≥5% to <50% of infiltrating tumor cells with a ratio ≥2.2 when using dual probes or ≥6 HER2 signals/cell using single probes^26^. Preclinical murine models of mixed HER2-expressing tumor cells have revealed that HER2 heterogeneity impacts response to anti-HER2 antibody therapy^27,28^. This may be explained in part because heterogeneity in HER2 expression may lead to variation in the cell cycle properties of tumors^29^. Clinically, the percentage of HER2-positive cells within the tumor, as well as IHC scores, correlate with response to anti-HER2 therapy^11,20^. Indeed, heterogenous HER2 expression is correlated with a high risk of relapse and resistance to chemotherapy and Trastuzumab in patients with HER2-positive breast cancer^27^. In a clinical trial that enrolled confirmed HER2-positive patients, HER2 ITH was assessed by central pathology review and defined as either: (1) HER2 positivity by ISH in > 5% and < 50% of tumor cells (i.e., CAP guideline) or (2) an area of the tumor that tested HER2 negative in at least one of the six areas evaluated per tumor^30^. HER2 ITH was determined to be a strong predictor of resistance to a dual-HER2-targeted therapy regimen (T-DM1 plus Pertuzumab), with no patients with cancers classified as heterogeneous experiencing a pCR^30^. This effect was also evident in subgroup analysis by HR status^30^. These data further support hormone receptor status as a possible driver of ITH in HER2+ breast cancer^31,32^. The infiltration of TILs has been shown to be inversely correlated with HR expression^33^, suggesting varied immune activity in HR+/HER2+ versus HR−/HER2+ cancers as contributing to differential response to HER2-targeted therapy^34^.

Methods to assess HER2 heterogeneity at a single-cell level across large populations of tumor cells may provide important information beyond the data from routine clinical IHC. Here, we used cyclic immunofluorescence (CyCIF), a microscopy platform for multiplex tissue imaging, to evaluate HER2 expression in a cohort of HER2-enriched tumors. With CyCIF, iterative four-channel imaging is performed (with each cycle involving different antibodies directly conjugated to fluorophores) from a single section of a formalin-fixed paraffin-embedded (FFPE) tumor specimen allowing the acquisition of data on 60 or more different antigens^35–37^. Images are then registered and stitched to generate a composite representation that is used for visualization and analysis^38,39^. Because CyCIF permits imaging across an entire tissue section, it is an appropriate method for evaluating the tumor and immune heterogeneity present in tumors and biopsies^35–37,40^. Given the ability of CyCIF to enable single-cell imaging analysis, we hypothesized that CyCIF imaging would support a better understanding of breast ITH. As with most new technologies that utilize immunostaining, appropriate antibody validation is key to reliable performance. Therefore, in this study, we first evaluated multiple commercially available fluorophore-conjugated antibodies directed against proteins commonly used to characterize breast carcinomas, including ER, PR, HER2, androgen receptor (AR), and p53. After assembling a qualified panel of antibodies, we performed single-cell multiplexed tissue imaging and analysis and identified tumor cell clusters that were associated with distinct clinical features, including heterogeneous HER2 expression. Single marker expression of HER2 ITH correlated with clinical outcome as previously described. Further, by using multiple tumor and immune markers, we derived heterogeneity scores and demonstrated that high heterogeneity measured through single-cell analysis may have predictive value for patients with poorer clinical outcomes.

## Results

### Qualifying antibodies for CyCIF

**Fig. 1:**
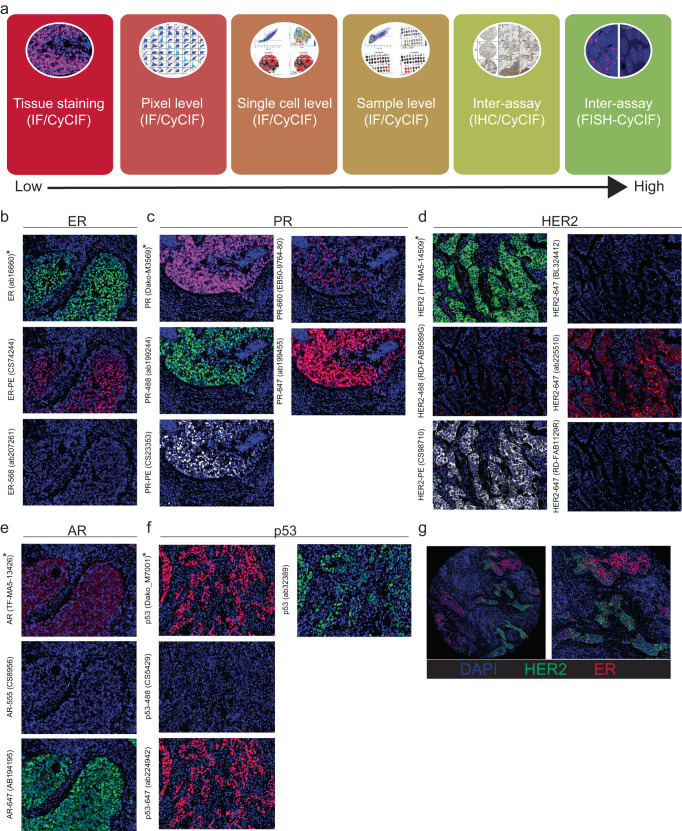
Overview of fluorescent CyCIF antibody qualification against antibodies used in the clinical laboratory. To qualify breast cancer-related antibodies HER2, ER, PR, AR, and p53, the BC03 tissue microarray (TMA), which represents 16 breast tumors in duplicate, was used. Multiple CyCIF antibodies were compared to a single antibody commonly used in clinical practice as a reference. **a** Schematic representation of the different levels of fluorescent antibody validation using the CyCIF method, starting from tissue staining (lowest level of validation) towards patient-level (highest level) inter-assay comparison (i.e., direct comparison of each patient tissue to itself between assays). **b**–**f** Representative CyCIF images of antibodies tested by CyCIF on the BC03 TMA. Asterisks indicate clinical antibodies (*) and qualified CyCIF antibodies (**) for each target. **g** Representative CyCIF image of HER2 (TF-MA5-14509; sp3) and ER (CS98710) staining, showing the majority of tumor cells are ER+, and some showing strong, membrane staining for HER2. Left image is a full TMA core (36× mag.); the right image corresponds to the left image (74× mag.).

Routine clinical testing of ER, PR, and HER2 is conducted in CLIA-certified laboratories that must document proficiency against pre-established criteria^41,42^. Recognizing the importance of having concordance between the results obtained from clinical testing and multiplexed tissue imaging, we first focused on testing the performance of multiple antibody clones against ER, PR, HER2, AR, and p53. To qualify these antibodies for use in CyCIF, we used a quantitative approach recently developed for assembling antibody panels for multiplexed tissue imaging assays (Fig. 1a)^43^. Single FFPE sections of human tissue were stained with 2 to 5 different commercially available, fluorophore-conjugated antibodies against the same protein target (Table 1), and the signal intensity from the different clones was compared. The performance of fluorophore-conjugated antibodies was evaluated against the clinical-grade antibodies used in practice by the Pathology Department at Brigham and Women’s Hospital (BWH)^44^.

**Table 1:** Clinical and CyCIF antibodies used in the study.

No.	Target	AB name	Vendor	Cat. no.	Performance by CyCIF	Notes	Selected for final panel?
ab1	AR	AR (AR441)	Thermo Fisher	TF-MA5-13426	*	Clinical	n/a
ab2	AR	AR-555	CST	8956	*	CyCIF	N
ab3	AR	AR-647	Abcam	AB194195	***	CyCIF	Y
ab1	ER	ER (sp1)	Abcam	ab16660	***	Clinical	n/a
ab2	ER	ER-PE	CST	74244	***	CyCIF	Y
ab3	ER	ER-568	Abcam	ab207261	**	CyCIF	N
ab4	ER	ER-647	Abcam	ab205851	**	CyCIF	N
ab1	HER2	HER2 (sp3)	Thermo Fisher	TF-MA5-14509	***	Clinical	n/a
ab2	HER2	HER2-488	R&D	RD-FAB9589G	**	CyCIF	N
ab3	HER2	HER2-PE	CST	98710	***	CyCIF	Y
ab4	HER2	HER2-647(BL)	Biolegend	BL324412	*	CyCIF	N
ab5	HER2	HER2-647	Abcam	ab225510	***	CyCIF	Y
ab6	HER2	HER2-647(RD)	R&D	RD-FAB1129R	**	CyCIF	N
ab1	p53	p53 (DO-7)	Abcam	Dako_M7001	***	Clinical	n/a
ab2	p53	p53-488	CST	5429	*	CyCIF	N
ab3	p53	p53-647	Abcam	ab224942	***	CyCIF	Y
ab4	p53	anti-p53	Abcam	ab32389	***	unconjugated	n/a
ab1	PR	PR (PgR636)	DAKO	Dako-M3569	***	Clinical	n/a
ab2	PR	PR-488	Abcam	ab199224	***	CyCIF	Y
ab3	PR	PR-647	Abcam	ab199455	***	CyCIF	Y
ab4	PR	PR-660	Ebioscience	EB50-9764-80	**	CyCIF	N
ab5	PR	PR-PE	CST	23353	**	CyCIF	N

Clinical antibodies are indicated as ab1. Qualified CyCIF antibodies are indicated by “Y” in the last column. Performance by CyCIF is ranked from 1 asterisk to 3 asterisks as shown.

*no signal.

**signal in some tissues, but no concordance with clinical antibodies.

***strong signal & show agreement with clinical antibodies.

Antibody testing was initially performed using a commercial tissue microarray (TMA; BC03), which included 32 samples, representing breast tumors from 16 patients arrayed in duplicate. CyCIF and corresponding clinical antibodies were applied to the same FFPE tissue following antigen retrieval using the standard CyCIF protocol as previously described^35–37^. Typically, CyCIF can accommodate unconjugated antibodies from different species (or isotypes) in the first cycle of staining, which are subsequently detected by indirect immunofluorescence using secondary antibodies conjugated to fluorophores. The clinical antibodies, which are often only available in unconjugated formulations, were therefore applied in the first cycle in unconjugated form. Fluorophore-conjugated CyCIF antibodies (i.e., primary antibodies conjugated directly to fluorophores) were used in subsequent cycles. Tables 1 and 2 detail the fluorophore-conjugated antibodies (referred to as “*CyCIF antibodies*”) used in the antibody qualification phase of this study. The clinical and CyCIF antibodies displayed expected staining patterns by CyCIF imaging when assessed by visual inspection (Fig. 1b–g), except for the clinical-grade AR antibody, which underperformed in the CyCIF assay compared to the CyCIF antibodies throughout the project (Fig. 1e).

**Table 2:** Antibody staining panels used for BC03 TMA.

Cycle #	BC03_A (PR/Ki67)		BC03_B (ER/p53)		BC03_C (AR/p53/Ki67)		BC03_D (HER2)	
Background	Hoechst1	Hoechst1	Hoechst1	Hoechst1	Hoechst1	Hoechst1	Hoechst1	Hoechst1
	FITC_1	A488	FITC_1	A488	FITC_1	A488	FITC_1	A488
	Cy3_1	A555	Cy3_1	A555	Cy3_1	A555	Cy3_1	A555
	Cy5_1	A647	Cy5_1	A647	Cy5_1	A647	Cy5_1	A647
2	Hoechst2	Hoechst2	Hoechst2	Hoechst2	Hoechst2	Hoechst2	Hoechst2	Hoechst2
	FITC_2	HER2 (TF-MA5-14509)	FITC_2	ER (ab16660)	FITC_2	p53 (ab32389)	FITC_2	HER2 (TF-MA5-14509)
	Cy3_2	14-3-3 (sc-629-G)	Cy3_2	14-3-3 (sc-629-G)	Cy3_2	14-3-3 (sc-629-G)	Cy3_2	14-3-3 (sc-629-G)
	Cy5_2	PR (Dako-M3569)	Cy5_2	p53 (Dako_M7001)	Cy5_2	AR (TF-MA5-13426)	Cy5_2	p53 (ab154036)
3	Hoechst3	Hoechst3	Hoechst3	Hoechst3	Hoechst3	Hoechst3	Hoechst3	Hoechst3
	FITC_3	PR-488 (ab199244)	FITC_3	PR-488 (ab199244)	FITC_3	p53-488 (CS5429)	FITC_3	HER2-488 (RD-FAB9589G)
	Cy3_3	PR-PE (CS23353)	Cy3_3	ER-PE (CS74244)	Cy3_3	AR-555 (CS8956)	Cy3_3	HER2-PE (CS98710)
	Cy5_3	PR-647 (ab199455)	Cy5_3	PR-660 (EB50-9764-80)	Cy5_3	AR-647 (AB194195)	Cy5_3	HER2-647 (BL324412)
4	Hoechst4	Hoechst4	Hoechst4	Hoechst4	Hoechst4	Hoechst4	Hoechst4	Hoechst4
	FITC_4	PR-488 (ab199244)	FITC_4	Ki67-488 (CS11882)	FITC_4	PR-488 (ab199244)	FITC_4	Ki67-488 (CS11882)
	Cy3_4	Ki67-570 (EB41-5699-82)	Cy3_4	ER-568 (ab207261)	Cy3_4	CK-570 (EB41-9003-82)	Cy3_4	CK-570 (EB41-9003-82)
	Cy5_4	PR-660 (EB50-9764-80)	Cy5_4	HER2-647 (RD-FAB1129R)	Cy5_4	p53-647 (ab224942)	Cy5_4	HER2-647 (ab225510)
5	Hoechst5	Hoechst5	Hoechst5	Hoechst5	Hoechst5	Hoechst5	Hoechst5	Hoechst5
	FITC_5	Ki67-488 (CS11882)	FITC_5	p53-488 (CS5429)	FITC_5	Ki67-488 (CST11882)	FITC_5	PR-488 (ab199244)
	Cy3_5	ER-PE (CS74244)	Cy3_5	CK-570 (EB41-9003-82)	Cy3_5	Ki67-570 (EB41-5699-82)	Cy3_5	CK-555 (CS3478)
	Cy5_5	Ki67-647 (CS12075)	Cy5_5	p53-647 (ab224942)	Cy5_5	Ki67-647 (BL350509)	Cy5_5	HER2-647 (RD-FAB1129R)
6	Hoechst6	Hoechst6	Hoechst6	Hoechst6	Hoechst6	Hoechst6	Hoechst6	Hoechst6
	FITC_6	p53-488 (CS5429)	FITC_6	HER2-488 (RD-FAB9589G)	FITC_6	HER2-488 (RD-FAB9589G)	FITC_6	p53-488 (CS5429)
	Cy3_6	ER-568 (ab207261)	Cy3_6	PR-PE (CS23353)	Cy3_6	HER2-PE (CS98710)	Cy3_6	ER-PE (CS74244)
	Cy5_6	ER-647 (ab205851)	Cy5_6	PR-647 (ab199455)	Cy5_6	HER2-647 (BL324412)	Cy5_6	AR-647 (AB194195)

**Fig. 2:**
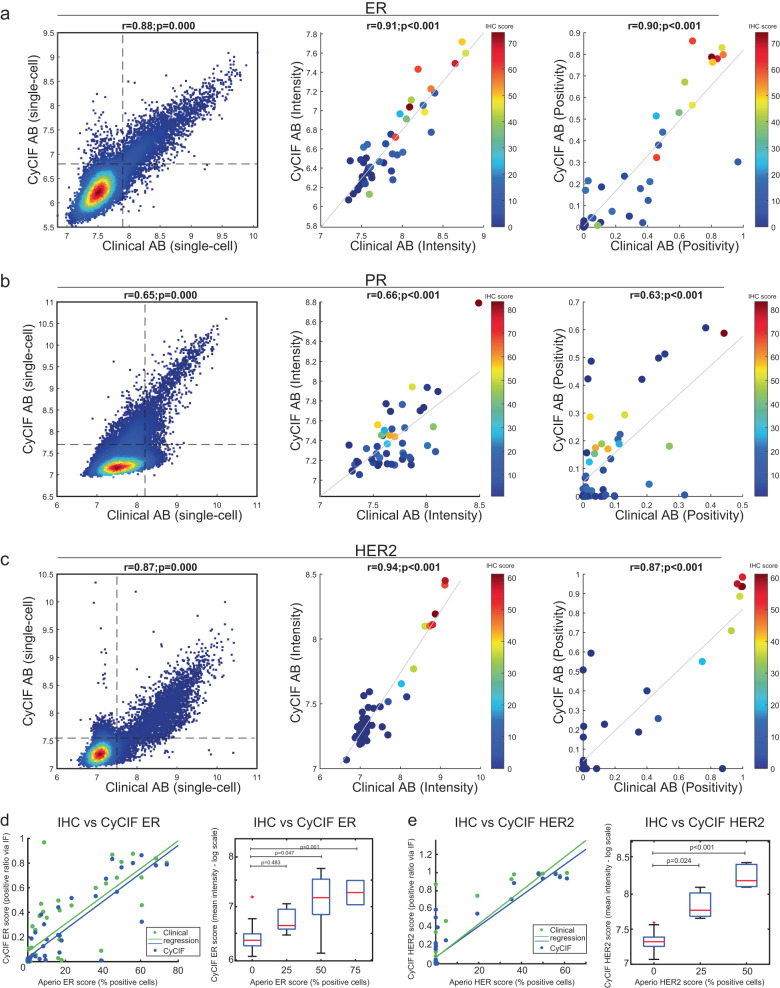
Core-to-core comparison of clinical and CyCIF antibodies against ER, PR, and HER2. To qualify breast cancer-related antibodies, the BC03 TMA, representing 16 breast tumors in duplicate was used. **a**–**c** CyCIF was performed using the qualified CyCIF antibody against a single antibody commonly used in clinical practice as a reference for ER (**a**), PR (**b**), and HER2 (**c**). The left graph depicts a single-cell dot-plot between the clinical clone on the *x* axis and the validated CyCIF antibody on the *y* axis. Each dot represents single-cell fluorescent intensity values from the two antibodies. Dashed lines indicate the gating cutoffs. The middle graph shows the corresponding mean log intensity of the core-to-core analysis of the clinical and CyCIF antibodies. The single-cell data were collected for individual TMA cores, with a binary gate applied to obtain the positive signal of each core (range from 0–1). The *X*- & *Y* axis represent the positive score calculated from either clinical or CyCIF antibodies, respectively. The right graph shows positivity scores (number of positive cells over total cells) for the clinical and CyCIF antibodies by TMA case. **d**, **e** Cross-assay comparison of the clinical and CyCIF antibodies analyzed by CyCIF compared to the clinical antibody analyzed by IHC using Aperio software for ER (**d**) and HER2 (**e**). Left, dot-plot representation of two different scores obtained from CyCIF and from IHC-Aperio. CyCIF of clinical (green dots) and CyCIF antibodies (blue dots) were used on the same section, while IHC was performed on a different section from the same TMA block. Each dot represents a single core from BC03 TMA. CyCIF scores are plotted on *y* axis as positive ratio of immunofluorescence, IHC scores on *x* axis are plotted as the percent of positive cells. Right graph, quantitative assessment of ER and HER2 IHC versus CyCIF staining. IHC scores by Aperio were used to stratify (0–24, 25–49, 50–74, 75–100) different TMA cores/cases, and the mean intensities of CyCIF antibody staining from each TMA core are shown using boxplot analysis. CyCIF antibodies: ER (CST 74244 S) and HER2 (ab225510).

**Fig. 3:**
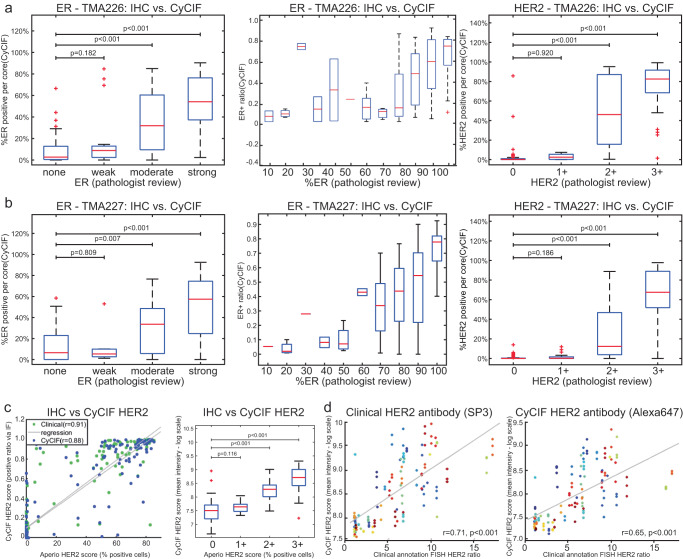
Inter-assay analysis of HER2 enriched TMAs (TMAs 226 and 227). Following the selection of qualified ER, PR, and HER2 antibodies, two HER2-enriched TMAs, which included 567 tissue cores (representing 189 patients in triplicate), were used to further qualify CyCIF antibodies. **a**, **b** Percent of ER+ and HER2+ cells assessed through CyCIF (*y* axis) is compared to the score assigned by a clinical pathologist (x-axis) for each TMA. **c** Cross-assay comparison of the HER2 clinical and CyCIF antibodies analyzed by CyCIF compared to the clinical antibody analyzed by IHC using Aperio software. Left, dot-plot represents two different scores obtained from CyCIF and one obtained from IHC-Aperio. CyCIF of clinical (green dots) and CyCIF (blue dots) antibodies were used on the same section, while IHC was done on a different section from the same TMA block. Each dot represents a single core from BC03 TMA. CyCIF scores are plotted on *y* axis as positive ratio of immunofluorescence, IHC scores on *x* axis plotted as percent of positive cells. Right, quantitative assessment HER2 IHC versus CyCIF staining. IHC scores by Aperio were used to stratify (0–24, 25–49, 50–74, 75–100) different TMA cores/cases, and the mean intensities of CyCIF antibody staining from each TMA core are shown using boxplot analysis. **d** Clinically annotated HER2 FISH scores against IF/CyCIF staining using the SP3 antibody (Pearson *r* = 0.71) and HER2 FISH scores against IF/CyCIF staining using the CyCIF antibody, ab225510 (Pearson *r* = 0.65). Individual patients are shown in different colors, in triplicate. The triplicate cores tend to cluster together, indicating minimal variation.

The CyCIF antibodies were next assessed against the clinical antibodies at multiple levels of analysis (Fig. 1a), including at the pixel-level (*pixel-by-pixel* comparison; Supplementary Fig. 1), and on a per-cell level (*cell-to-cell* comparison; Supplementary Fig. 2). After we had selected a single high performing CyCIF antibody for each of the targets (ones that performed at least as well as the clinical-grade antibody in the pixel and cell level comparisons), we then assessed the signal intensity values acquired at the level of individual tissue cores (*sample-to-sample* level comparisons; Fig. 2, Supplementary Fig. 3). In addition, inter-assay comparisons of antibody performance between CyCIF and IHC (Fig. 3, Supplementary Fig. 4) and between CyCIF and HER2 FISH (in HER2-positive breast tumors) was performed to provide orthogonal qualification (Fig. 3).

The pixel-level analysis involved computing fluorescence intensity values for each antibody at a single pixel resolution and then performing a pixel-to-pixel correlation between the antibodies of the same target. This analysis revealed strong concordance between most CyCIF antibodies and their corresponding clinical antibody. Random sampling of 5000 pixels from 32 samples revealed Pearson correlation coefficients generally ranging from 0.70 to 0.97 (Supplementary Fig. 1). As expected, the DNA/Hoechst signal was not correlated with the epitope-specific signal generated by the antibodies (Supplementary Fig. 1). The pixel-level data of fluorescent intensity also allowed us to evaluate the dynamic range for each antibody revealing that most antibodies could capture and discriminate both low- and high-expressing cells (Supplementary Fig. 1, box plots). While most CyCIF antibodies performed well, some had poor correlation to other antibodies, including the clinical antibody. For example, the HER2 CyCIF ab4 had suboptimal performance compared to the clinical antibody, as demonstrated by a narrow dynamic range and lower sensitivity (Supplementary Fig. 1C, D). DNA/Hoechst was used as a reference and showed a wide dynamic range, as expected.

Multi-channel whole slide imaging data is typically segmented to identify single cells, and the staining intensity in each channel is computed on a per-cell basis^38^. Therefore, we next performed cell-to-cell comparisons of the signal acquired from the clinical antibody for each target to each of the CyCIF antibodies (Supplementary Fig. 2A–C, image on the left). Briefly, cells were segmented as described in the methods, and 5000 random cells were computationally isolated and analyzed from the 32 samples. Similar to the pixel-level comparisons, the cell-to-cell analysis revealed that the signal generated by most CyCIF and clinical antibodies was highly correlated (Supplementary Fig. 2, middle plot, intensity of each cell is plotted in log scale) and demonstrated a wide dynamic range indicating that these antibodies could detect both cells with low and high antigen expression (Supplementary Fig. 2A–E, boxplot on the right). The HER2 CyCIF ab4 that had not performed well in the pixel analysis similarly performed poorly in the cell-to-cell analysis with a narrow dynamic range and lower correlation coefficient with the clinical antibody compared to the correlation coefficient of other CyCIF antibodies versus the clinical antibody (Supplementary Fig. 2C).

### Testing qualified CyCIF antibodies

Top performing CyCIF antibodies were identified based on the highest correlation with the clinical antibody and other CyCIF antibodies, highest performance in signal-to-noise ratio assessment, wide dynamic range, and best overall performance upon visual inspection (Table 1). The performance of the selected CyCIF antibodies was then tested again against the clinical antibodies. The BC03 TMA was stained with both the qualified CyCIF panel and clinical antibodies, and sample-level analysis was performed (core-to-core comparisons). The single-cell data was collected for individual TMA cores, and the mean log intensity of the signal for each antibody was used to calculate correlations. These analyses revealed concordance with R values of 0.91 for ER, and 0.94 for HER2 between the clinical and CyCIF antibodies (Fig. 2a–c and Supplementary Fig. 3A, B, middle plot). Of note, the clinical PR antibody (PgR636) was less sensitive than the conjugated CyCIF PR antibody, resulting in a minor discrepancy in the correlation between cores, likely because the CyCIF antibody identified more PR+ cells. After binary gating using a 2-component Gaussian Mixture Model (GMM), there was excellent core-to-core correlation between the positivity ratio (the number of positive cells divided by total cells of each core; ranging from 0~1) for the ER, PR, HER2, and p53 antibodies (Fig. 2a–c, Supplementary Fig. 3, far right graphs). A poor correlation was observed, however, for the AR antibodies due to the poor performance of the clinical-grade AR antibody in the CyCIF assay (Supplementary Fig. 3A). This can be explained given that the clinical antibody was selected for clinical testing based on its performance in IHC, which uses a protocol that differs from the CyCIF protocol. Indeed, we confirmed that the clinical AR antibody performed as expected by IHC (Supplementary Fig. 3C) but failed to work well in CyCIF due to a high background signal (Supplementary Fig. 3d, ab1).

In the initial evaluation, clinical-grade antibodies had been used as unconjugated reagents in the CyCIF assay. In the subsequent validation step, we compared the performance of the CyCIF antibodies against the clinical-grade antibodies used in standard IHC (i.e., cross-assay comparison between CyCIF and IHC). For this comparison, CyCIF was performed on single FFPE sections from TMA BC03 using both the CyCIF and clinical antibodies, and IHC was performed in the BWH Pathology Department Laboratory using the clinical antibodies on a serial section from the same TMA (Supplementary Fig. 4A). The IHC using the clinical antibodies was scored in two different ways: (i) using Aperio digital pathology software (recorded as percent positive cells) and (ii) by microscopic inspection by two pathologists (according to a clinical scoring schema). The Aperio IHC score of the clinical antibody was then compared to the positive ratio of the two different antibodies (the CyCIF and the clinical antibodies) as measured by CyCIF (Fig. 2d, e; Supplementary Fig. 4B, C). The Aperio IHC scores (% positive cells) from the clinical antibodies are shown on the x-axis and are plotted in two ways: (i) against itself in the CyCIF assay (green dots) and (ii) against the CyCIF antibody (blue dots). The clinical antibody IHC scores (x-axis) by Aperio were used to stratify TMA cores/cases, and the mean intensities of CyCIF staining of both the clinical and CyCIF antibodies (y-axis) from each TMA core are shown using boxplot analysis (Fig. 2d, e and Supplementary Fig. 4B, C, boxplot). ER and HER2 scoring of the CyCIF data had a high correlation (clinical antibody vs. CyCIF antibody) with Aperio IHC scoring (Fig. 2d, e). As expected, the clinical AR antibody by IHC was not correlated to itself when used in the CyCIF assay (green dots) but the clinical IHC analysis demonstrated a high correlation to the CyCIF AR antibody (blue; *r* = 0.74; Supplementary Fig. 4B) supporting the use of the CyCIF AR antibody. We also found high correlation between the clinical-grade p53 antibody and a CyCIF p53 antibody on core-level analysis (Supplementary Fig. 4C). Across the study we found that ‘mean fluorescence intensity’ (rather than the positive ratio via CyCIF) correlates better with the Aperio IHC score. This may in part be because Aperio scoring reflects mean expression across cells in the tissue.

The correlation of CyCIF to semiquantitative scoring of the IHC by two pathologists was then assessed. Scores from two pathologists for ER and HER2 IHC were highly correlated with the Aperio IHC scoring (Supplementary Fig. 4D–H). Our analysis of the TMA cores revealed some discrepancies with the results available from the vendor of the TMA, which may be attributable to the fact that the vendor scoring was not performed on immediate serial sections and no information was provided regarding the antibodies that had been used by the vendor (Supplementary Fig. 4G, H).

The cross-assay comparison was then extended to include two HER2-enriched TMAs (TMA226 and 227) from a cohort of samples from patients who were diagnosed with their primary breast cancer between March 1995 and November 2005 and subsequently treated at the Dana-Farber/Brigham and Women’s Cancer Center (Table 3). The tissues were annotated with clinical data, including the results of HER2 FISH that was performed as part of clinical care (Table 3). TMA226 and 227 include 567 tissue cores from 189 tumors arrayed in triplicate^45,46^. CyCIF was performed on a single slide from each TMA, and serial sections were used for ER and HER2 IHC. The CyCIF images were analyzed to identify the percent of marker-positive cells out of the total keratin-positive tumor cell population. IHC was scored in two ways: (i) by a pathologist according to CAP guidelines for ER (none, weak, moderate, strong) and percent of positive cells; and HER2 (0, 1+, 2+, 3+) and (ii) using Aperio software as a percent of positive cells. The CyCIF and IHC pathology scores were highly correlated for ER and HER2 (Fig. 3a, b) as were the CyCIF and Aperio scores of HER2 (Fig. 3c). We found high correlation between HER2 copy number (as measured by HER2 FISH analysis) and the expression of HER2 protein as determined through CyCIF using both the clinical and CyCIF antibodies (Pearson *r* = 0.71 and 0.65, respectively; Fig. 3d). Individual cores from the TMA are plotted in Fig. 3d colored by patient. While we observed differences between cores from the same patient, they largely clustered together, indicating that each sample resembles the larger tissue. Taken together, these analyses identified fluorophore-conjugated CyCIF antibodies, which compare favorably to widely used clinical antibodies.

**Table 3:** Clinical annotation of TMAs 226, 227, 240.

Code	AGE	Stage	Histology	Grade	ER_IHC	PR_IHC	HER2_IHC	HER2_FISH	Recurrence	Vital
Her2-001	40.7	I	Invasive Ductal With EIC	III (High)	Positive	Positive	3 +	Not performed	No	Dead
Her2-002	51.8	I	Invasive Ductal	III (High)	Negative	Negative	3 +	Not performed	No	Alive
Her2-003	54.3	IIA	Invasive Ductal	II	Positive	Positive	3 +	Not performed	No	Alive
Her2-004	66.7	IIA	Invasive Lobular	III (High)	Positive	Positive	High Pos	Not performed	No	Alive
Her2-005	41.9	I	Invasive Ductal	III (High)	Negative	Negative	3 +	Not performed	No	Dead
Her2-006	69.9	IIIC	Invasive Ductal	II	Positive	Positive	3 +	Positive	Yes	Dead
Her2-007	38.0	IIB	Invasive Ductal	II	Positive	Negative	High Pos	Not performed	Yes	Alive
Her2-008	37.6	I	Invasive Ductal With EIC	II	Positive	Positive	3 +	Not performed	No	Alive
Her2-009	46.9	I	Invasive Ductal	III (High)	Positive	Positive	3 +	Not performed	Yes	Alive
Her2-010	55.6	IIB	Invasive Ductal With EIC	II	Positive	Negative	3 +	Not performed	No	Alive
Her2-011	66.3	I	Invasive Ductal	II	Positive	Negative	2 +	Not performed	No	Alive
Her2-012	57.1	IIIC	Invasive Lobular	III (High)	Negative	Negative	2 +	Negative	Yes	Dead
Her2-013	41.3	IIA	Invasive Ductal	III (High)	Negative	Positive	3 +	Not performed	No	Alive
Her2-014	32.1	I	Invasive Ductal	III (High)	Positive	Positive	3 +	Not performed	No	Alive
Her2-015	56.1	IIA	Invasive Ductal	I (Low)	Positive	Positive	2 +	Not performed	No	Alive
Her2-016	30.2	IIA	Invasive Ductal With EIC	III (High)	Negative	Positive	3 +	Not performed	No	Alive
Her2-017	52.9	IIA	Invasive Ductal	II	Positive	Positive	2 +	Not performed	No	Alive
Her2-018	56.2	IIA	Invasive Ductal	II	Positive	Positive	2 +	Not performed	No	Alive
Her2-019	49.8	IIB	Invasive Ductal With EIC	II	Positive	Positive	High Pos	Not performed	No	Alive
Her2-020	73.6	IIIA	Invasive Lobular	II	Positive	Positive	3 +	Positive	No	Dead
Her2-021	40.3	IIB	Invasive Ductal With EIC	III (High)	Negative	Negative	3 +	Not performed	No	Alive
Her2-022	51.8	IIA	Invasive Ductal With EIC	II	Positive	Positive	3 +	Not performed	Yes	Alive
Her2-023	46.5	I	Invasive Ductal	III (High)	Positive	Negative	3 +	Not performed	No	Alive
Her2-024	31.8	IIB	Invasive Ductal With EIC	III (High)	Positive	Negative	3 +	Not performed	No	Dead
Her2-025	53.7	IIB	Invasive Ductal	III (High)	Positive	Positive	3 +	Not performed	No	Alive
Her2-026	55.5	IIB	Invasive Lobular	II	Positive	Positive	2 +	Negative	Yes	Dead
Her2-027	54.4	IIB	Invasive Ductal	III (High)	Negative	Negative	3 +	Not performed	Yes	Alive
Her2-028	43.0	IIA	Invasive Ductal	III (High)	Positive	Positive	3 +	Not performed	No	Alive
Her2-029	58.3	I	Invasive Ductal	II	Positive	Positive	Low Pos	Not performed	No	Alive
Her2-030	32.4	IIB	Invasive Ductal	III (High)	Positive	Positive	3 +	Not performed	Yes	Alive
Her2-031	92.1	Can’t Stage	Invasive Ductal and Lobular	III (High)	Negative	Negative	3 +	Not performed	No	Dead
Her2-034	38.6	IIB	Invasive Ductal	III (High)	Negative	Negative	3 +	Not performed	No	Alive
Her2-035	45.6	IIA	Invasive Ductal With EIC	III (High)	Positive	Positive	3 +	Not performed	Yes	Dead
Her2-036	53.4	I	Invasive Ductal	III (High)	Positive	Negative	3 +	Positive	No	Alive
Her2-037	54.6	IIIA	Invasive Ductal	III (High)	Negative	Negative	3 +	Positive	Yes	Dead
Her2-038	42.6	0	Invasive Ductal	II	Positive	Positive	High Pos	Positive	No	Alive
Her2-039	45.0	IIA	Invasive Ductal With EIC	III (High)	Positive	Positive	2 +	Negative	No	Alive
Her2-040	49.0	I	Invasive Ductal With EIC	III (High)	Negative	Negative	3 +	Not performed	No	Alive
Her2-041	35.0	IIB	Invasive Ductal With EIC	III (High)	Positive	Positive	2 +	Not performed	No	Alive
Her2-042	46.7	I	Invasive Ductal	III (High)	Negative	Positive	High Pos	Not performed	Yes	Alive
Her2-043	53.7	IIIA	Invasive Lobular	II	Positive	Positive	2 +	Negative	No	Alive
Her2-044	57.4	IV	Invasive Lobular	III (High)	Negative	Negative	3 +	Not performed	Yes	Alive
Her2-045	53.1	IIB	Invasive Ductal	III (High)	Negative	Negative	3 +	Not performed	No	Alive
Her2-046	50.9	IIB	Invasive Ductal	II	Positive	Positive	High Pos	Not performed	No	Alive
Her2-047	62.0	I	Invasive Ductal	II	Positive	Positive	3 +	Not performed	No	Alive
Her2-048	55.6	IIIB	Invasive Ductal	III (High)	Negative	Negative	3 +	Positive	Yes	Dead
Her2-049	52.8	IIA	Invasive Ductal	II	Positive	Negative	3 +	Not performed	No	Alive
Her2-050	60.9	IIB	Invasive Ductal With EIC	II	Positive	Positive	2 +	Negative	Yes	Dead
Her2-051	51.3	I	Invasive Ductal	II	Positive	Positive	3 +	Not performed	No	Alive
Her2-052	60.7	I	Invasive Ductal With EIC	II	Negative	Negative	3 +	Not performed	No	Alive
Her2-053	50.0	Can’t Stage	Invasive Ductal	II	Positive	Positive	1 +	Not performed	No	Alive
Her2-054	63.4	I	Invasive Ductal	III (High)	Negative	Negative	2 +	Not performed	No	Dead
Her2-055	68.2	IIIA	Invasive Ductal and Lobular	III (High)	Negative	Negative	2 +	Not performed	No	Dead
Her2-056	56.8	IIA	Invasive Ductal	III (High)	Positive	Positive	3 +	Positive	No	Alive
Her2-057	34.8	IIA	Invasive Ductal	III (High)	Positive	Negative	3 +	Not performed	No	Alive
Her2-058	70.7	IIB	Invasive Lobular	III (High)	Positive	Positive	3 +	Not performed	No	Alive
Her2-059	72.1	IV	Invasive Ductal	II	Negative	Negative	2 +	Not performed	Yes	Dead
Her2-060	40.0	IIA	Invasive Ductal With EIC	III (High)	Positive	Positive	3 +	Not performed	No	Alive
Her2-061	63.1	IIA	Invasive Ductal With EIC	III (High)	Negative	Negative	2 +	Negative	No	Alive
Her2-062	58.9	IIIB	Invasive Ductal and Lobular	II	Negative	Negative	2 +	Not performed	No	Alive
Her2-063	72.2	IIA	Invasive Ductal	II	Negative	Negative	3 +	Not performed	No	Alive
Her2-064	38.4	IIB	Invasive Ductal	III (High)	Positive	Positive	2 +	Not performed	No	Alive
Her2-065	53.7	IIA	Invasive Ductal	I (Low)	Positive	Positive	3 +	Not performed	No	Alive
Her2-066	33.2	IIB	Invasive Ductal and Lobular	III (High)	Positive	Positive	2 +	Positive	No	Alive
Her2-067	41.4	IIB	Invasive Ductal With EIC	III (High)	Positive	Positive	2 +	Negative	No	Alive
Her2-068	48.0	IIB	Invasive Ductal and Lobular	II	Positive	Positive	2 +	Negative	No	Alive
Her2-069	49.2	IIA	Invasive Ductal With EIC	III (High)	Positive	Positive	3 +	Not performed	No	Alive
Her2-070	32.5	IIA	Invasive Ductal	III (High)	Negative	Negative	3 +	Not performed	No	Alive
Her2-071	71.5	I	Invasive Ductal	II	Positive	Positive	2 +	Not performed	No	Dead
Her2-072	44.4	I	Invasive Ductal and Lobular	III (High)	Positive	Positive	3 +	Positive	No	Alive
Her2-073	56.0	I	Invasive Ductal	I (Low)	Positive	Positive	2 +	Negative	No	Alive
Her2-074	79.8	I	Invasive Ductal	I (Low)	Positive	Negative	2 +	Not performed	No	Alive
Her2-075	60.7	I	Invasive Ductal With EIC	II	Negative	Negative	3 +	Not performed	No	Alive
Her2-076	59.1	IIB	Invasive Ductal With EIC	III (High)	Positive	Negative	High Pos	Not performed	No	Alive
Her2-077	68.3	IIA	Invasive Ductal With EIC	II	Positive	Positive	High Pos	Not performed	No	Alive
Her2-078	83.3	IIB	Invasive Ductal With EIC	III (High)	Negative	Negative	3 +	Not performed	Yes	Dead
Her2-079	55.4	IIA	Invasive Ductal	III (High)	Negative	Negative	3 +	Not performed	Yes	Dead
Her2-080	69.5	IIB	Invasive Lobular	II	Positive	Negative	2 +	Not performed	Yes	Dead
Her2-081	30.3	IIA	Invasive Ductal With EIC	II	Positive	Positive	3 +	Not performed	No	Alive
Her2-082	46.6	IIA	Invasive Ductal With EIC	III (High)	Positive	Positive	3 +	Not performed	No	Alive
Her2-083	38.3	I	Invasive Ductal With EIC	III (High)	Negative	Negative	3 +	Not performed	Yes	Alive
Her2-084	48.3	IIA	Invasive Ductal With EIC	III (High)	Positive	Positive	3 +	Not performed	No	Dead
Her2-085	46.1	I	Invasive Ductal With EIC	I (Low)	Positive	Positive	2 +	Negative	No	Alive
Her2-086	56.7	IIA	Invasive Lobular	I (Low)	Positive	Positive	2 +	Negative	No	Alive
Her2-087	37.2	I	Invasive Ductal	II	Negative	Negative	3 +	Not performed	No	Alive
Her2-088	41.8	IIA	Invasive Ductal With EIC	III (High)	Positive	Positive	3 +	Not performed	Yes	Alive
Her2-089	50.6	IIA	Invasive Ductal With EIC	III (High)	Positive	Positive	3 +	Not performed	No	Alive
Her2-090	59.8	I	Invasive Ductal	III (High)	Positive	Negative	2 +	Not performed	No	Alive
Her2-091	78.6	IIA	Invasive Ductal	II	Negative	Negative	3 +	Not performed	No	Alive
Her2-092	41.5	IIA	Invasive Ductal	II	Positive	Positive	2 +	Positive	Yes	Alive
Her2-093	32.3	IIB	Invasive Ductal and Lobular	III (High)	Positive	Positive	2 +	Positive	No	Alive
Her2-094	43.1	IIA	Invasive Ductal	III (High)	Positive	Positive	3 +	Not performed	No	Alive
Her2-095	49.2	IIIA	Invasive Ductal With EIC	III (High)	Positive	Negative	3 +	Not performed	No	Alive
Her2-096	59.2	IIIA	Invasive Ductal With EIC	III (High)	Positive	Positive	2 +	Positive	No	Alive
Her2-097	61.1	IIB	Invasive Ductal With EIC	III (High)	Positive	Negative	3 +	Not performed	Yes	Alive
Her2-098	53.1	IIA	Invasive Ductal	III (High)	Positive	Positive	2 +	Negative	No	Alive
Her2-101	48.2	I	Invasive Ductal and Lobular	III (High)	Positive	Positive	3 +	Not performed	No	Alive
Her2-102	54.3	I	Invasive Ductal	II	Positive	Negative	3 +	Not performed	Yes	Dead
Her2-103	47.4	I	Invasive Ductal	II	Positive	Positive	2 +	Not performed	No	Alive
Her2-104	52.1	I	Invasive Ductal	III (High)	Positive	Positive	2 +	Not performed	No	Alive
Her2-105	44.8	IIB	Invasive Ductal	III (High)	Positive	Positive	3 +	Not performed	Yes	Dead
Her2-106	58.2	IIIA	Invasive Ductal	III (High)	Positive	Positive	2 +	Not performed	No	Alive
Her2-107	79.5	I	Invasive Ductal With EIC	III (High)	Positive	Negative	3 +	Not performed	No	Dead
Her2-108	61.4	I	Invasive Ductal	III (High)	Negative	Negative	3 +	Not performed	Yes	Alive
Her2-109	82.9	IIA	Invasive Ductal and Lobular	III (High)	Positive	Positive	2 +	Not performed	No	Dead
Her2-110	54.9	IIIB	Invasive Ductal With EIC	II	Positive	Positive	2 +	Not performed	No	Alive
Her2-111	51.8	IIIA	Invasive Ductal With EIC	III (High)	Positive	Positive	2 +	Negative	Yes	Dead
Her2-112	53.2	I	Invasive Ductal and Lobular	I (Low)	Positive	Negative	3 +	Not performed	No	Alive
Her2-113	64.5	I	Invasive Ductal	II	Positive	Positive	3 +	Not performed	No	Alive
Her2-114	39.4	I	Invasive Ductal With EIC	III (High)	Negative	Negative	3 +	Not performed	Yes	Alive
Her2-115	60.9	I	Invasive Ductal	III (High)	Negative	Negative	2 +	Positive	No	Alive
Her2-116	47.7	I	Invasive Ductal With EIC	III (High)	Positive	Negative	3 +	Not performed	Yes	Dead
Her2-117	39.1	IIA	Invasive Ductal and Lobular	II	Positive	Positive	3 +	Not performed	No	Alive
Her2-118	57.8	I	Invasive Ductal	I (Low)	Positive	Positive	Low Pos	Not performed	No	Alive
Her2-119	42.7	IIA	Invasive Ductal	II	Positive	Negative	3 +	Not performed	No	Alive
Her2-120	59.6	IIB	Invasive Ductal and Lobular	II	Positive	Positive	2 +	Not performed	Yes	Dead
Her2-121	49.0	IIA	Invasive Ductal	III (High)	Positive	Negative	3 +	Not performed	No	Alive
Her2-122	55.9	IIIA	Invasive Ductal and Lobular	II	Positive	Negative	2 +	Not performed	No	Alive
Her2-123	40.2	II	Invasive Ductal	III (High)	Positive	Negative	3 +	Not performed	No	Dead
Her2-124	47.8	I	Invasive Ductal With EIC	II	Positive	Positive	2 +	Not performed	No	Alive
Her2-125	51.6	IIA	Invasive Ductal	II	Positive	Positive	3 +	Positive	No	Alive
Her2-126	53.5	II	Invasive Ductal	III (High)	Negative	Negative	Negative	Not performed	No	Alive
Her2-127	55.9	I	Invasive Ductal	II	Positive	Positive	2 +	Negative	No	Dead
Her2-128	26.8	IIIA	Invasive Ductal	III (High)	Positive	Positive	3 +	Positive	No	Alive
Her2-129	62.0	I	Invasive Ductal	I (Low)	Positive	Positive	2 +	Not performed	No	Dead
Her2-130	49.5	I	Invasive Ductal With EIC	II	Positive	Positive	High Pos	Not performed	No	Alive
Her2-131	54.5	I	Invasive Ductal	II	Positive	Positive	2 +	Negative	No	Alive
Her2-132	42.5	IIA	Invasive Ductal With EIC	III (High)	Positive	Positive	3 +	Not performed	No	Alive
Her2-133	46.2	I	Invasive Ductal With EIC	II	Positive	Positive	2 +	Not performed	No	Alive
Her2-134	45.7	I	Invasive Ductal	III (High)	Negative	Negative	3 +	Not performed	No	Alive
Her2-135	59.8	I	Invasive Ductal	III (High)	Positive	Negative	2 +	Not performed	No	Alive
Her2-136	45.6	IIA	Invasive Ductal	III (High)	Positive	Positive	2 +	Not performed	No	Alive
Her2-137	41.0	IIA	Invasive Ductal With EIC	II	Positive	Positive	3 +	Not performed	No	Alive
Her2-138	55.5	I	Invasive Ductal and Lobular	I (Low)	Positive	Positive	3 +	Positive	No	Alive
Her2-139	63.1	IIB	Invasive Ductal	II	Positive	Positive	3 +	Not performed	No	Dead
Her2-140	45.2	I	Invasive Ductal With EIC	II	Positive	Positive	2 +	Not performed	Yes	Alive
Her2-141	61.9	IIA	Invasive Ductal	III (High)	Negative	Negative	High Pos	Not performed	No	Alive
Her2-142	42.5	IIA	Invasive Ductal and Lobular	II	Positive	Positive	3 +	Negative	No	Alive
Her2-143	37.6	I	Invasive Ductal With EIC	III (High)	Positive	Positive	High Pos	Not performed	No	Dead
Her2-144	50.0	I	Invasive Ductal	III (High)	Negative	Negative	3 +	Not performed	No	Alive
Her2-145	56.4	I	Invasive Ductal	II	Positive	Positive	2 +	Not performed	No	Alive
Her2-146	38.0	IIIA	Invasive Ductal With EIC	III (High)	Positive	Positive	3 +	Not performed	No	Alive
Her2-147	57.3	I	Invasive Ductal	III (High)	Positive	Positive	3 +	Not performed	No	Alive
Her2-148	45.7	IIA	Invasive Ductal With EIC	I (Low)	Positive	Negative	2 +	Not performed	No	Alive
Her2-149	46.1	IIA	Invasive Ductal	II	Positive	Positive	2 +	Negative	No	Alive
Her2-150	87.8	II	Invasive Lobular	II	Positive	Positive	Negative	Not performed	No	Dead
Her2-151	49.9	I	Invasive Ductal With EIC	II	Positive	Positive	2 +	Not performed	No	Alive
Her2-152	36.4	IIA	Invasive Ductal	III (High)	Negative	Negative	3 +	Not performed	No	Alive
Her2-153	41.8	IIB	Invasive Ductal and Lobular	II	Positive	Positive	High Pos	Not performed	No	Alive
Her2-154	67.2	IIB	Invasive Ductal	II	Negative	Negative	3 +	Not performed	Yes	Alive
Her2-155	64.2	IIA	Invasive Ductal	III (High)	Positive	Positive	2 +	Negative	No	Alive
Her2-156	45.7	IIA	Invasive Ductal and Lobular	II	Positive	Positive	3 +	Not performed	No	Alive
Her2-157	72.7	I	Invasive Ductal	I (Low)	Positive	Positive	2 +	Not performed	No	Alive
Her2-158	41.4	I	Invasive Ductal and Lobular	II	Positive	Positive	3 +	Not performed	No	Alive
Her2-159	56.9	IIA	Invasive Ductal	II	Positive	Positive	3 +	Not performed	Yes	Dead
Her2-160	47.0	I	Invasive Ductal	II	Positive	Positive	2 +	Negative	No	Alive
Her2-161	64.2	IIIA	Invasive Lobular	I (Low)	Positive	Positive	3 +	Not performed	No	Dead
Her2-162	71.9	I	Invasive Ductal	II	Positive	Positive	2 +	Not performed	Yes	Alive
Her2-163	58.4	IIB	Invasive Ductal With EIC	II	Positive	Positive	2 +	Positive	No	Alive
Her2-164	41.5	IIB	Invasive Ductal	III (High)	Negative	Negative	3 +	Not performed	Yes	Alive
Her2-165	44.9	IIB	Invasive Ductal	II	Positive	Positive	2 +	Not performed	No	Alive
Her2-166	38.0	IIB	Invasive Ductal With EIC	II	Positive	Positive	2 +	Negative	No	Alive
Her2-167	47.5	IIA	Invasive Ductal	III (High)	Positive	Negative	3 +	Not performed	Yes	Alive
Her2-168	38.2	IIB	Invasive Ductal	III (High)	Positive	Negative	High Pos	Not performed	Yes	Dead
Her2-169	39.5	I	Invasive Ductal With EIC	II	Positive	Positive	3 +	Not performed	No	Alive
Her2-170	63.2	IIIB	Invasive Ductal and Lobular	I (Low)	Positive	Positive	2 +	Negative	Yes	Dead
Her2-171	82.6	I	Invasive Ductal With EIC	II	Positive	Positive	2 +	Not performed	No	Dead
Her2-172	76.4	I	Invasive Ductal and Lobular	II	Positive	Positive	2 +	Not performed	No	Dead
Her2-173	59.9	IIB	Invasive Ductal	III (High)	Positive	Positive	2 +	Not performed	No	Alive
Her2-174	49.6	IIB	Invasive Ductal With EIC	III (High)	Positive	Positive	High Pos	Not performed	No	Alive
Her2-175	36.1	I	Invasive Ductal With EIC	II	Positive	Positive	3 +	Not performed	Yes	Alive
Her2-176	53.7	IIA	Invasive Ductal	III (High)	Positive	Negative	3 +	Not performed	Yes	Dead
Her2-177	43.4	IIA	Invasive Ductal	I (Low)	Positive	Positive	2 +	Negative	No	Alive
Her2-178	40.7	I	Invasive Ductal and Lobular	II	Positive	Positive	2 +	Negative	No	Alive
Her2-179	60.4	IIB	Invasive Ductal With EIC	III (High)	Negative	Negative	3 +	Not performed	No	Alive
Her2-180	45.6	IIB	Invasive Ductal and Lobular	III (High)	Positive	Positive	3 +	Not performed	No	Dead
Her2-181	42.3	IIB	Invasive Ductal	III (High)	Negative	Negative	2 +	Positive	No	Alive
Her2-182	63.4	IIA	Invasive Ductal With EIC	II	Positive	Positive	2 +	Negative	No	Alive
Her2-183	40.3	I	Invasive Ductal	II	Positive	Positive	High Pos	Negative	Yes	Alive
Her2-184	48.9	IIB	Invasive Ductal With EIC	III (High)	Negative	Negative	3 +	Positive	Yes	Dead
Her2-185	86.3	IIA	Invasive Ductal With EIC	II	Positive	Positive	2 +	Not performed	No	Alive
Her2-186	49.3	IIA	Invasive Ductal	III (High)	Positive	Positive	2 +	Not performed	No	Alive
Her2-187	65.6	IIIA	Invasive Ductal	III (High)	Positive	Positive	2 +	Negative	Yes	Dead
Her2-188	59.9	I	Invasive Ductal	II	Positive	Positive	2 +	Negative	No	Alive
Her2-190	61.7	I	Invasive Ductal	I (Low)	Positive	Positive	High Pos	Negative	No	Alive
Her2-191	82.3	IIB	Invasive Ductal	III (High)	Negative	Negative	3 +	Not performed	No	Alive
Her2-192	69.4	IV	Invasive Ductal	III (High)	Negative	Negative	High Pos	Not performed	Yes	Dead
Her2-193	40.0	IIB	Invasive Ductal With EIC	II	Negative	Negative	3 +	Not performed	No	Alive
Her2-194	48.4	IIA	Invasive Ductal	II	Positive	Positive	3 +	Not performed	Yes	Dead
TN-001	62.6	IIA	Invasive Ductal	III (High)	Negative	Negative	Negative	Not performed	No	
TN-002	56.2	I	Invasive Ductal	III (High)	Negative	Negative	Negative	Not performed	No	
TN-003	37.0	IIB	Invasive Ductal	III (High)	Negative	Negative	Negative	Not performed	No	
TN-004	53.2	IIIB	Invasive Ductal	III (High)	Negative	Negative	Negative	Not performed	No	
TN-005	50.3	IIIA	Invasive Ductal	II	Negative	Negative	Negative	Not performed	Yes	
TN-006	47.5	IIIB	Invasive Ductal	III (High)	Negative	Negative	Negative	Not performed	No	
TN-007	59.9	IIIB	Invasive Ductal and Lobular	III (High)	Negative	Negative	Negative	Not performed	No	
TN-008	47.6	IIB	Invasive Ductal	III (High)	Negative	Negative	Negative	Not performed	No	
TN-009	62.0	I	Invasive Ductal With EIC	III (High)	Negative	Negative	Negative	Not performed	No	
TN-010	44.2	IIA	Invasive Ductal With EIC	II	Negative	Negative	1 +	Negative	No	
TN-011	48.9	I	Invasive Ductal	III (High)	Negative	Negative	Negative	Not performed	No	
TN-012	40.4	IIB	Invasive Ductal	III (High)	Negative	Negative	Negative	Not performed	No	
TN-013	43.6	I	Invasive Ductal	III (High)	Negative	Negative	Negative	Not performed	No	
TN-014	58.5	IV	Invasive Ductal	III (High)	Negative	Negative	Negative	Negative	Yes	
TN-015	43.5	IIA	Invasive Ductal	III (High)	Negative	Negative	1 +	Negative	No	
TN-016	64.4	IIB	Invasive Ductal With EIC	III (High)	Negative	Negative	1 +	Not performed	No	
TN-017	76.8	IIIA	Invasive Ductal	III (High)	Negative	Negative	Negative	Not performed	No	
TN-018	48.7	IIB	Invasive Ductal With EIC	III (High)	Negative	Negative	Negative	Not performed	No	
TN-019	42.2	IIA	Invasive Ductal	III (High)	Negative	Negative	Negative	Not performed	No	
TN-020	42.0	II	Invasive Ductal	III (High)	Negative	Negative	Negative	Not performed	No	
TN-021	54.0	IIA	Invasive Ductal	III (High)	Negative	Negative	1 +	Not performed	No	
TN-022	57.2	I	Invasive Ductal and Lobular	III (High)	Negative	Negative	Negative	Not performed	No	
TN-023	78.6	I	Invasive Ductal	III (High)	Negative	Negative	Negative	Not performed	No	
TN-024	50.9	IIA	Invasive Ductal	III (High)	Negative	Negative	Not performed	Not performed	No	
TN-025	53.9	IIIB	Invasive Ductal	III (High)	Negative	Negative	Negative	Not performed	Yes	
TN-026	30.1	IIB	Invasive Ductal	III (High)	Negative	Negative	Negative	Not performed	No	
TN-027	53.0	IIA	Invasive Ductal and Lobular	II	Positive	Positive	1 +	Not performed	Yes	
TN-028	38.4	I	Invasive Ductal	III (High)	Negative	Negative	1 +	Not performed	No	
TN-029	67.1	I	Invasive Ductal	III (High)	Negative	Negative	Negative	Not performed	Yes	
TN-030	33.1	I	Invasive Ductal With EIC	III (High)	Negative	Negative	Negative	Not performed	No	
TN-031	79.5	IV	Invasive Ductal	III (High)	Positive	Positive	Negative	Not performed	Yes	
TN-032	55.2	IIIB	Invasive Ductal and Lobular	III (High)	Negative	Negative	Negative	Not performed	Yes	
TN-033	67.6	0	Invasive Ductal	III (High)	Negative	Negative	1 +	Not performed	No	
TN-034	44.0	IIA	Invasive Ductal	III (High)	Negative	Negative	1 +	Not performed	Yes	
TN-035	58.9	IIIA	Invasive Ductal	III (High)	Negative	Negative	Negative	Not performed	Yes	
TN-036	28.6	I	Medullary	III (High)	Negative	Negative	Negative	Not performed	No	
TN-037	39.5	I	Invasive Ductal	III (High)	Negative	Negative	Negative	Not performed	No	
TN-038	74.4	IIA	Invasive Ductal	II	Negative	Negative	Negative	Not performed	No	
TN-039	57.6	IIB	Invasive Ductal	III (High)	Negative	Negative	1 +	Not performed	Yes	
TN-040	36.1	I	Invasive Ductal With EIC	III (High)	Negative	Negative	Negative	Not performed	No	
TN-041	35.3	I	Invasive Ductal	III (High)	Negative	Negative	Negative	Not performed	Yes	
TN-042	51.3	IIB	Invasive Ductal	III (High)	Negative	Negative	Negative	Not performed	No	
TN-043	52.6	IIA	Invasive Ductal	III (High)	Negative	Negative	1 +	Not performed	No	
TN-044	46.3	IIA	Invasive Ductal	III (High)	Negative	Negative	1 +	Not performed	No	
TN-045	68.6	I	Invasive Ductal With EIC	III (High)	Negative	Negative	Negative	Not performed	No	
TN-046	35.7	IIA	Invasive Ductal	III (High)	Negative	Negative	Negative	Not performed	No	
TN-047	58.2	IIA	Invasive Ductal	III (High)	Negative	Negative	Negative	Not performed	No	
TN-048	48.0	I	Invasive Ductal	III (High)	Negative	Negative	Negative	Not performed	No	
TN-049	51.4	I	Invasive Ductal and Lobular	III (High)	Negative	Negative	1 +	Not performed	No	
TN-050	52.0	IIA	Invasive Ductal	III (High)	Negative	Negative	Negative	Not performed	No	
TN-051	56.6	IIA	Invasive Ductal	III (High)	Negative	Negative	Negative	Not performed	No	
TN-052	72.8	IIA	Invasive Ductal	III (High)	Negative	Negative	Negative	Not performed	No	
TN-053	56.1	IIB	Invasive Ductal	III (High)	Negative	Negative	Negative	Negative	Yes	
TN-054	50.5	IIA	Invasive Ductal	III (High)	Negative	Negative	Negative	Not performed	No	
TN-055	57.5	IIA	Invasive Ductal	II	Negative	Negative	Negative	Not performed	Yes	
TN-056	59.1	I	Invasive Ductal	III (High)	Negative	Negative	Negative	Not performed	No	
TN-057	54.7	I	Invasive Ductal	III (High)	Negative	Negative	Negative	Not performed	No	
TN-058	40.9	IIB	Invasive Ductal	III (High)	Negative	Negative	Negative	Not performed	No	
TN-059	68.2	I	Invasive Ductal	II	Negative	Negative	Negative	Not performed	No	
TN-060	52.1	I	Invasive Ductal	II	Negative	Negative	Negative	Not performed	No	
TN-061	66.9	IIB	Invasive Ductal	III (High)	Negative	Negative	Negative	Not performed	Yes	
TN-062	49.2	IIB	Invasive Ductal	III (High)	Negative	Negative	1 +	Not performed	No	
TN-063	79.6	IV	Invasive Ductal	III (High)	Negative	Negative	Negative	Not performed	Yes	
TN-064	64.3	IIB	Invasive Ductal	III (High)	Negative	Negative	Negative	Not performed	No	
TN-065	67.3	IIB	Invasive Ductal	III (High)	Negative	Negative	Negative	Not performed	No	
TN-066	56.8	IIB	Invasive Ductal With EIC	III (High)	Negative	Negative	Negative	Not performed	No	
TN-067	51.2	IIA	Invasive Ductal	II	Negative	Negative	1 +	Not performed	No	
TN-068	68.7	IIB	Invasive Ductal	III (High)	Negative	Negative	1 +	Not performed	No	
TN-069	54.4	Can’t Stage	Invasive Ductal	III (High)	Negative	Negative	Negative	Not performed	No	
TN-070	37.2	IIIA	Invasive Ductal	III (High)	Negative	Negative	Negative	Not performed	No	
TN-071	59.6	I	Invasive Ductal	III (High)	Negative	Negative	Negative	Not performed	Yes	
TN-072	28.9	IIB	Invasive Ductal	III (High)	Negative	Negative	Negative	Negative	No	
TN-073	57.2	I	Invasive Ductal	III (High)	Negative	Negative	Negative	Not performed	Yes	
TN-074	64.5	II	Invasive Ductal and Lobular	III (High)	Negative	Negative	Negative	Not performed	No	
TN-075	51.2	I	Invasive Ductal	III (High)	Negative	Negative	Negative	Not performed	No	
TN-076	51.9	I	Invasive Ductal	III (High)	Negative	Negative	Negative	Not performed	No	
TN-077	68.8	I	Invasive Ductal and Lobular	III (High)	Negative	Negative	Negative	Not performed	No	
TN-078	56.0	I	Invasive Ductal	III (High)	Negative	Negative	1 +	Not performed	No	
TN-079	67.6	I	Invasive Ductal	III (High)	Negative	Negative	1 +	Not performed	No	
TN-080	53.5	I	Invasive Ductal	II	Negative	Negative	Negative	Not performed	No	
TN-081	42.9	IIA	Invasive Ductal	III (High)	Negative	Negative	Negative	Not performed	No	
TN-082	37.2	IIA	Invasive Ductal	III (High)	Negative	Negative	Negative	Not performed	No	
TN-083	42.7	IIA	Invasive Ductal	III (High)	Negative	Negative	Negative	Not performed	No	
TN-084	44.4	IIA	Invasive Ductal	III (High)	Negative	Negative	2 +	Negative	No	
TN-085	78.4	IIB	Invasive Ductal	III (High)	Negative	Negative	1 +	Not performed	Yes	
TN-086	84.2	I	Adenocystic	II	Negative	Negative	2 +	Not performed	No	
TN-087	41.9	I	Invasive Ductal	III (High)	Negative	Negative	Negative	Not performed	No	
TN-088	56.0	I	Invasive Ductal	III (High)	Negative	Negative	Negative	Not performed	No	
TN-089	47.7	I	Invasive Ductal	III (High)	Negative	Negative	Negative	Not performed	No	

### A qualified antibody panel accurately assigned single cells based on clinical annotation

Having established a qualified CyCIF antibody panel (Supplementary Fig. 5; Table 4), we next characterized the ITH of breast tumors at a single-cell level. CyCIF was performed on the two HER2-enriched TMAs (TMA226 and 227) and an additional TMA that was enriched for triple-negative breast cancer samples (TMA240). Together, the TMAs included 834 total breast tumor cores from 278 unique patients, including HER2+ (regardless of HR status; *n* = 158, 57%), HR+/HER2− (*n* = 31, 11%) and HR−/HER2− (TNBC; *n* = 89, 32%) (Tables 3, 5). A total of 512,699 single cells were segmented, and fluorescence intensity values were computed on a per-cell basis (Table 6). While the full data set was used for analysis, the data from 50,000 randomly selected cells was used for visualization in the t-distributed stochastic neighbor embedding (t-SNE), which projects the integrated staining intensity for each cell onto two dimensions preserving the high-dimensional relationships between the makers (Supplementary Fig. 6). Tumor cells (i.e., Keratin positive) single cells clustered according to the clinical annotation that was extracted from the clinical database of the corresponding tumor (HER2+ [regardless of HR status], HR+/HER2− and HR−/HER2−) and, as expected, the immune cells were randomly distributed (Supplementary Fig. 6A). Keratin positive (Supplementary Fig. 6B) tumor cells expressed combinations of ER, PR and HER2 as expected in partially overlapping patterns (Supplementary Fig. 6C). Ki67 was expressed in subsets of the HR+/HER2+, HR−/HER2+ and HR−/HER2− tumor cells. AR was co-expressed in a subset of HR+ tumors and in a subset of HR−/HER2− tumor cells. p53 was predominantly expressed in HR−/HER2− tumor cells (Supplementary Fig. 6D). Keratin negative cells were positive for CD45 and/or CD68 and a subset of those expressed PD-L1 (Supplementary Fig. 6E).

**Table 4:** Qualified antibody staining panel.

Cycle #			
Background	DAPI_1		
	FITC_1	A488	Background
	Cy3_1	A555	Background
	Cy5_1	A647	Background
2	DAPI_2		
	FITC_2	HER2 (TF-MA5-14509)	Thermo (Rabbit)
	Cy3_2	53BP1 (Bethy A303-906A)	Bethyl (Goat)
	Cy5_2	p53 (Dako_M7001)	DAKO (DO-7 IgG2b)
3	DAPI_3		
	FITC_3	PR-488 (ab199244)	Abcam
	Cy3_3	ER-PE (CS74244)	CST
	Cy5_3	PR-647 (ab199455)	Abcam
4	DAPI_4		
	FITC_4	Ki67-488 (CS11882)	CST
	Cy3_4	HER2-PE (CS98710)	CST
	Cy5_4	AR-647 (AB194195)	Abcam
5	DAPI_5		
	FITC_5	CD45-488 (FAB1430G)	R&D (2D1 clone)
	Cy3_5	CK-570 (EB41-9003-82)	Ebio
	Cy5_5	p53-647 (ab224942)	Abcam
6	DAPI_6		
	FITC_6	p53-FITC (Bio645803)	Biolegend (DO-7)
	Cy3_6	PD-L1-555 (AB206616)	Abcam
	Cy5_6	HER2-647 (ab225510)	Abcam
7	DAPI_7		
	FITC_7	CD68-488(CST24850)	CST
	Cy3_7	pRb-555(CST8957)	CST
	Cy5_7	PD-L1-647(CST15005)	CST

**Table 5:** Total number of cases by ER/PR/HER2 status.

	ER+PR+HER2+	ER+PR-HER2+	ER-PR+HER2+	HR−/HER2+	HR+/HER2-	HR−/HER2-	TOTAL CASES
TMAs: 226+227	88	27	3	39	29	3	189
TMA: 240	0	0	0	1	2	86	89
Total number of cases	**88**	**27**	**3**	**40**	**31**	**89**	**278**
Percent 226+227	47%	14%	2%	21%	15%	2%	
Percent 240	0%	0%	0%	1%	2%	97%	
Total percent	32%	10%	1%	14%	11%	32%	
TMA226 and 227	HER2 enriched						
TMA240	TNBC						

Bold values indicate the summation of rows 1 and 2.

**Table 6:** Total number of single cells analyzed per subtype of breast cancer.

		# single cells analyzed
Group 1	HER2+	201,601
Group 2	HR+	94,237
Group 3	TNBC	216,861
TOTAL		512,699

### A qualified CyCIF antibody panel reveals distinct clusters of cancer cells in HER2+ breast cancer

**Fig. 4:**
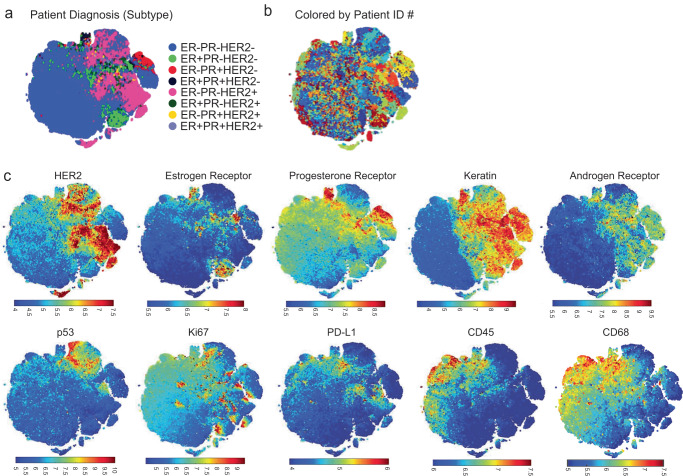
Overview of single-cell data from HER2+ enriched breast tumors. Following the selection of qualified ER, PR, and HER2 CyCIF antibodies, the expression of selected antibodies was evaluated at a single-cell level in 567 HER2+ invasive breast cancer samples from 189 patients, and t-Distributed Stochastic Neighbor Embedding (t-SNE) is shown as a distribution of all single cells. **a** Selected markers were used to plot single cells. **b** t-SNE in **a** is shown colored by patient ID. **c** Visualization of markers within t-SNE plots.

Given that the qualified antibody panel accurately assigned single cells based on clinical annotation, we performed a deeper analysis focusing on the two TMAs enriched with HER2+ tumors (567 tissue cores from 189 patients, a total of 201,601 single cells analyzed; Table 6). The tumor cells from the HER2 enriched cases were analyzed at the single-cell level, and single cells were clustered by their patterns of ER, PR, and HER2 expression (Fig. 4a). When the t-SNE was colored by a patient identifier (Fig. 4b), we observed a substantial degree of ITH for ER, PR and HER2 expression. In general, the tumors were enriched for HER2 expression as expected (Fig. 4c).

**Fig. 5:**
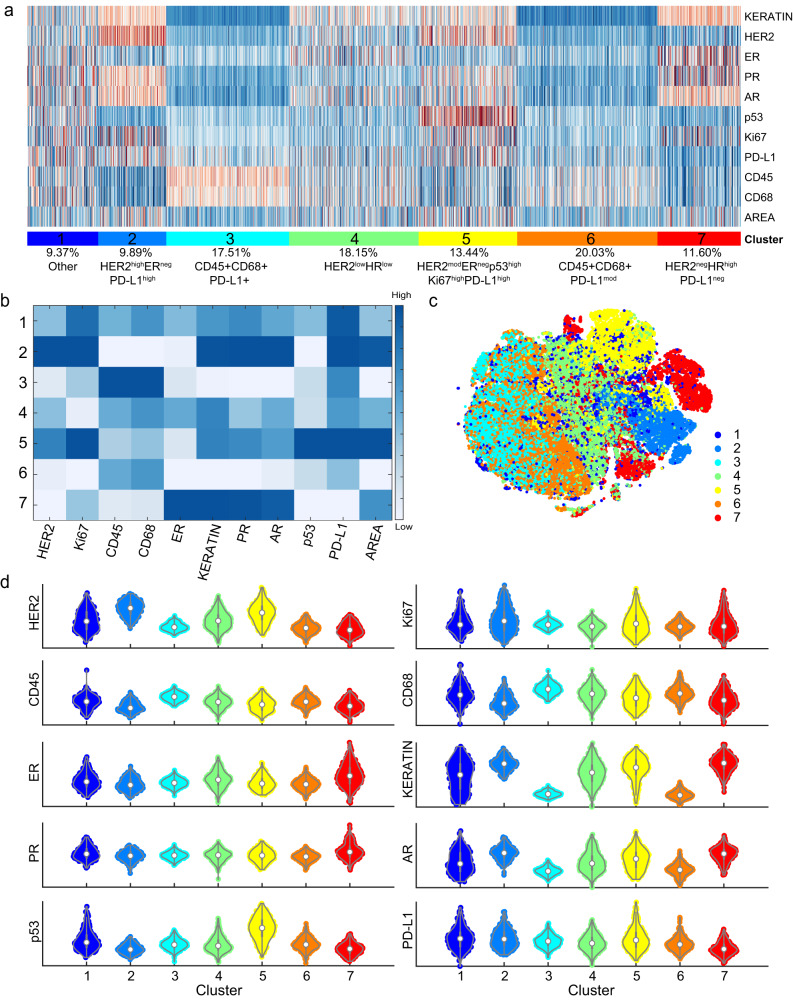
CyCIF single-cell analysis of HER2+ breast cancer reveals tumor populations with heterogenous HER2 expression. Following the selection of qualified ER, PR, HER2, AR, and p53 CyCIF antibodies, the expression of selected antibodies was evaluated at a single-cell level in 567 HER2+ invasive breast cancer samples, representing 189 patients. **a** Single-cell clustering is shown, and **b** median expression for each antigen across each cluster is shown. Relative expression of HER2 is designated as high, moderate (mod), low, and negative among clusters 2, 5, 4, and 7, respectively. Tumor clusters are defined as: cluster 2 (HER2^high^ER^neg^PR^pos^AR^pos^PD-L1^high^Ki67^pos^), cluster 5 (HER2^mod^ER^neg^PR^pos^AR^pos^p53^high^PD-L1^pos^Ki67^pos^), and cluster 4 (HER2^low^ER^pos^PR^low^AR^pos^)). One cluster was HER2-negative (cluster 7 (HER2^neg^ER^high^PR^high^AR^high^)). Cluster 3 and 6 represent immune/stromal populations as characterized by the expression of the leukocyte marker CD45. Cluster 1 has heterogeneous expression of most proteins and, therefore, did not form a distinct population of cells. Area in A and B refers to the nuclear area of segmented cells. **c** The 7 cell clusters are visualized using t-SNE. **d** Volcano plots of expression of each marker by cluster.

Clustering of all the single-cell (tumor and non-tumor cells) revealed 7 distinct clusters, including 4 tumor and 2 immune/stromal populations (Fig. 5a–d). Clusters 2, 4, 5, and 7 represented the tumor cells as defined by the expression of keratin. Among the 4 tumor populations, three were HER2+ and displayed different levels of HER2 expression relative to each other high, moderate and low: cluster 2 (HER2^high^ER^neg^PR^pos^AR^pos^PD-L1^high^Ki67^pos^), cluster 5 (HER2^mod^ER^neg^PR^pos^AR^pos^p53^high^PD-L1^pos^Ki67^pos^) and cluster 4 (HER2^low^ER^pos^PR^low^AR^pos^)). One cluster was HER2-negative (cluster 7 (HER2^neg^ER^high^PR^high^AR^high^)). Volcano plot analysis reveals heterogenous expression of markers across clusters (Fig. 5d). Clusters 3 and 6 represent an immune population as characterized by expression of the leukocyte marker CD45 and macrophage marker CD68, suggesting these are macrophages. Cluster 1 had heterogeneous expression of most proteins and, therefore, did not correspond to a distinct population of cells. We revealed that there was a low expression of HER2 and moderate expression of Keratin through the violin plot analysis (Fig. 5d) and that 69.89% of the cells had some Keratin positivity, 36.14% Her2 positivity, and 34.05% were double positive, overlapping with the single populations; therefore, it is likely a tumor cell population that also contains non-tumor cells within the cluster. The use of additional antibodies against other immune cells, endothelial, fibroblast, and other tumor markers would likely increase the ability to cluster additional cells into appropriate classes. Taken together, these analyses revealed the presence of substantial HER2 ITH in breast tumors at a single-cell level that may have implications for clinical care.

### CyCIF reveals ITH of HER2+ breast cancer

**Fig. 6:**
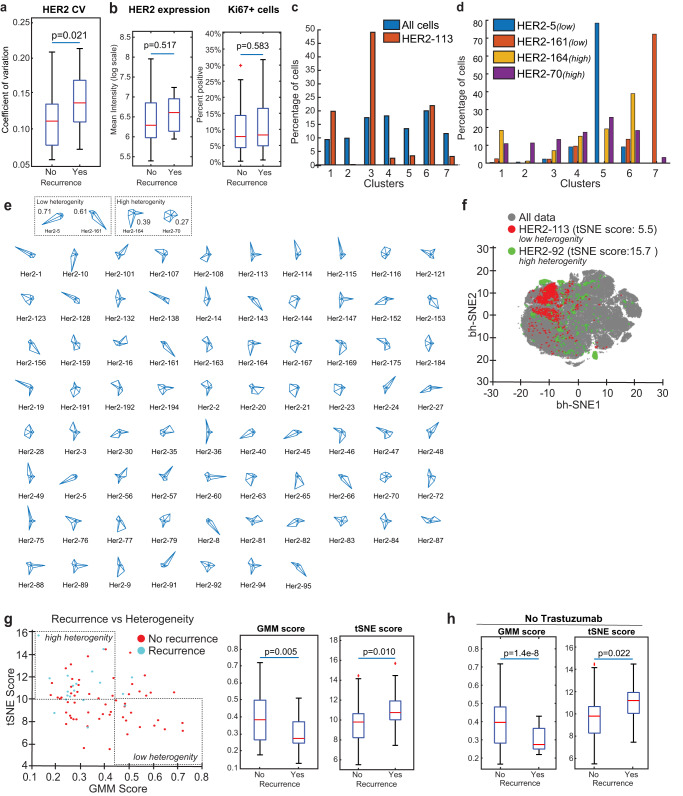
HER2 Heterogeneity scores derived from clustering analysis reveal correlation to clinical outcome. Following the selection of qualified ER, PR, HER2, AR, and p53 CyCIF antibodies, the expression of selected antibodies was evaluated at a single-cell level in 567 HER2+ invasive breast cancer samples, representing 189 patients. Tissues from HER2+ patients (*n* = 77) in which there were at least 500 cells pooled from the triplicate cores were used for ITH analysis. **a** HER2 expression was analyzed in single cells, and the coefficient of variation (C.V.) among patients was plotted (*y* axis) by recurrence status. **b** HER2 and Ki67 mean intensity expression measured by CyCIF. **c** Distribution of cells across all clusters (blue) and HER2 core number 113 (orange) and **d** representative tumor with low (HER2-5 and HER2-161) and high (HER2-164 and HER2-170) heterogeneity. **e** HER2 heterogeneity scores were generated by identifying cells from each tissue mapped to the entire t-SNE. A larger boundary corresponds with higher diversity. **f** Samples that have equal distribution of each cluster have high heterogeneity and are diamond-shaped in the boundary mapping. **g** GMM and t-SNE scores reveal an association with recurrence. **h** Patients treated with Trastuzumab were removed from the GMM and t-SNE score analysis.

Tumors with high HER2 ITH have been shown to be more resistant to HER2-targeted therapy, and recent data from clinical trials have implicated HER2 ITH in determining clinical outcome^30^. We evaluated HER2 expression in individual cells in tissue samples from 77 unique patients (triplicate cores; *n* = 231, from the cohort that was clinically defined as HER2+ and had at least 500 cells pooled from the triplicate cores) by association with recurrence data obtained from clinical records. The interpatient variation of HER2 expression as measured by the coefficient of variation in single cells revealed that higher heterogeneity in individual patients correlated with recurrence, as expected (Fig. 6a). The mean intensity expression of HER2 did not correlate with recurrence, nor did expression of Ki67, both measured by CyCIF, indicating that using single parameters of expression may not be sufficient in evaluating the tumor due the complexity of tumor heterogeneity (Fig. 6b).

It is unknown how the complexity of the tumor as a whole influences ITH and clinical outcome. Therefore, we then sought to characterize the ITH of HER2+ tumors beyond the expression of HER2 using the same 77 patients with triplicate cores. To do this, we developed two metrics to score the ITH of cell types, which we derived from (i) the GMM clusters and (ii) t-SNE representations of the CyCIF data. The GMM score is a population-level estimation of the heterogeneity of cell-type composition. Clustering all single cells from the cohort into 7 different subpopulations using GMM (Fig. 6c, d) allowed us to determine the GMM score for each sample, which reflects how the cell-type composition of the sample differs from the cell-type composition of the entire cohort. In the HER2 enriched cohort, the 7 clusters contained between 7–20% of the cells (Figs. 5c and 6d, blue bar). We observed that individual patient samples were enriched for cells derived from some clusters more than others. For example, the distribution of cells from core HER2-113 across the 7 GMM clusters is shown in Fig. 6c, orange bars, and reveals over-representation of Cluster 3 in that tumor, whereas other samples comprised a more even distribution of clusters and therefore had a more heterogenous composition (e.g., HER2-164 and HER2-70; Fig. 6d). HER2-5 and HER2-161 are more enriched in a single cluster, therefore, less heterogeneous (Fig. 6d). To visualize the composition of the samples, we generated glyph plots (Fig. 6e) and calculated the Euclidean distance from the mean distribution of all samples (see methods) to generate GMM scores. A high GMM score represents low heterogeneity, while a low GMM score represents heterogenous cell compositions (Supplementary Table 1). Next, we generated t-SNE scores which are derived directly from the single-cell data in high-dimensional space (Fig. 6f). Unlike the GMM score, the t-SNE score is not based on separating cells into different clusters, instead it uses the distance and spread of each single-cell in t-SNE space. A wider distribution of single cells for any given sample in t-SNE space represents tumors with high ITH, while tumors with low ITH have a more localized distribution (Fig. 6f).

To evaluate the potential clinical significance of ITH, we utilized the GMM and t-SNE scores along with recurrence data obtained from clinical records. The GMM and t-SNE scores revealed differences between patients who experienced recurrence versus those who did not (Fig. 6g). Since the HER2 enriched TMA cases are from patients who were diagnosed with their primary breast cancer between March 1995 and November 2005 and adjuvant Trastuzumab was not approved by the FDA until 2006, patients primarily received chemotherapy without anti-HER2 therapy (Table 7). To unify the analysis, we removed the small fraction of patients who did receive Trastuzumab (*n* = 10) and performed the analysis again with the remaining 67 patients (Table 7) and found the GMM correlation with recurrence as well as the t-SNE score correlation with recurrence followed a similar association as with the full data set (Fig. 6h).

**Table 7:** Treatment of HER+ patient cohort (TMAs 226 and 227).

Treatment	# pts in TMAs 226 and 227 (*n* = 189)	# pts in ITH cohort (*n* = 77)
Chemotherapy	33	18
Hormone therapy	25	6
Chemotherapy + hormone therapy	85	33
Chemotherapy + trastuzumab	6	4
Chemo + hormone + trastuzumab	12	6
n/a	28	10

We assessed additional associations with clinical data, including ER and PR status, clinical stage, age, and tumor grade, all extracted from the clinical data, as well as Ki67 expression derived from CyCIF analysis. In some cases, adjacent categories were combined when there were low numbers of patients for each category (Table 8A). Interestingly, none of these features were significantly associated with recurrence (Table 8B). We then fit two models, one with GMM score and the other with t-SNE score with the clinical features and found that both GMM and t-SNE scores were significantly associated with time to recurrence and among the other clinical features examined, only clinical stage (III-IV vs I) was significantly associated in both Model 1 (GMM score; *p* = 0.03; Table 8C) and Model 2 (t-SNE score; *p* = 0.049; Table 8C). Taken together, this work suggests that high ITH as measured through single-cell analysis, may be linked to poorer clinical outcomes.

**Table 8:** Association of clinical data.

A						
	*N* = 77					
Final stage						
I	27					
II	1					
IIA	28					
IIB	15					
IIIA	4					
IIIB	1					
IV	1					
Stage N						
1	27					
2	44					
3	5					
4	1					
Tumor grade						
I	3					
II	23					
III	51					
**B**						
		*N* = 77	GMM score (median, IQR)		t-SNE score (median, IQR)	
	ER			*P* value		*P* value
	Negative	24 (31.2)	0.4 (0.3–0.5)	0.42	10 (8.2–10.8)	0.59
	Positive	53 (68.8)	0.3 (0.3–0.4)		10.3 (8.7–11.3)	
	PR					
	Negative	36 (46.8)	0.3 (0.3–0.5)	0.37	9.9 (8.3–10.9)	0.21
	Positive	41 (53.2)	0.3 (0.1–0.7)		10.4 (8.8–11.4)	
	Clinical stage					
	I	27 (35.1)	0.4 (0.3–0.5)	0.19	9.7 (8.2–10.9)	0.47
	II	44 (57.1)	0.3 (0.3–0.4)		10.3 (8.8–11.4)	
	III–IV	6 (7.8)	0.4 (0.3–0.5)		9.6 (8.6–10.4)	
	Age (median, range)	48 (27–82)	pho = −0.05	0.69	pho = −0.16	0.17
	Ki67 (median, IQR)	0.1 (0.1–0.2)	pho = 0.02	0.88	pho = −0.14	0.23
	Tumor grade					
	I–II	26 (33.8)	0.3 (0.3–0.5)	0.94	10 (8.3–11)	0.57
	III (High)	51 (66.2)	0.3 (0.3–0.5)		10.2 (8.8–11.3)	
**C**		Hazard ratio (95% CI)	*P* value			
	Model 1 - GMM score					
	GMM	0.01 (0–0.77)	**0.04**			
	ER	0.38 (0.1–1.43)	0.15			
	PR	1.21 (0.30–4.93)	0.79			
	Clinical Stage (II vs I)	0.79 (0.27–2.30)	0.66			
	Clinical Stage (III–IV vs I)	7.13 (1.17–43.38)	**0.03**			
	Age	0.99 (0.95–1.03)	0.65			
	ki67	0.99 (0–133.4)	1			
	Tumor grade (high vs other)	1.75 (0.47–6.5)	0.4			
	Model 2 - t-SNE Score					
	t-SNE	1.41 (1.1–1.8)	**0.006**			
	ER	0.38 (0.09–1.65)	0.2			
	PR	1.02 (0.22–4.83)	0.98			
	Clinical Stage (II vs I)	0.78 (0.26–2.32)	0.66			
	Clinical Stage (III–IV vs I)	6.26 (1.01–38.80)	**0.049**			
	Age	0.99 (0.95–1.03)	0.6			
	ki67	2.63 (0.02–373.04)	0.7			
	Tumor grade (high vs other)	1.54 (0.41–5.82)	0.53			

Bold values indicate statistically significant values.

## Discussion

This study is the first to evaluate the performance of antibodies routinely used clinically to analyze breast cancers in a highly multiplexed imaging platform such as CyCIF that enables single-cell analysis across an entire tissue sample. We developed a panel of qualified antibodies against common breast cancer markers that show excellent concordance with clinical antibodies routinely used in CLIA-certified labs. We then used the qualified antibodies along with other cell states and immune markers to perform CyCIF. Using a HER2-enriched cohort of 567 tissue cores from 189 patients, we performed clustering analysis of 201,601 single cells. Clustering analysis allowed an unbiased approach to inform our understanding of how HER2 heterogeneity relates to other relevant cancer markers. Heterogenous expression of HER2 expression among individual patients correlated with recurrence. This has been previously reported using IHC analysis, but we report it here for the first time using single-cell analysis (Fig. 6a). Further, we identified 4 keratin positive tumor cell clusters that varied by HER2 expression levels relative to each other (high, moderate, low and negative). These clusters further varied with respect to other breast cancer-specific markers such as ER, PR, AR, and p53, as well as PD-L1. Importantly, we revealed that ITH correlates with clinical outcome.

Clustering of single cells from tumors using CyCIF revealed new classifications of HER2 heterogenous breast tumors. Indeed, we revealed that clusters 2 and 5 had high to moderate expression of HER2, PR, AR, and PD-L1but were negative for ER. Cluster 5 had high expression of p53, whereas cluster 2 was negative for p53. Cluster 4 consisted of a population of HER2^low^-expressing cells as well as low expression of ER, PR, and AR and heterogenous expression of PD-L1. Cluster 7 represented a HER2^neg^ER^pos^ population of tumor cells, which was also positive for PR and AR, and negative for p53 and PD-L1. ER+ tumors are generally associated with low tumor-infiltrating lymphocytes (TILs)^47^, and up-regulation of PD-L1 in the tumor has been shown to be driven by interferon-gamma production by CD8+T cells^48^. Therefore, the HER2^neg^ER^pos^ tumor Cluster 7 may represent an immunologically cold tumor environment indicated by the absence of PD-L1. ASCO/CAP acknowledges the spatial heterogeneity of HER2 staining as “clustered”, “mosaic” and “scattered”. These non-clonal patterns are more frequent in cases that are 2+. Our patient cohort had a limited sample size of HER2-low tumors, and therefore, we were not able to assess spatial heterogeneity among HER2-low tumors. However, a major advantage of the CyCIF technology is the ability to perform spatial analysis, and therefore, further investigation of spatial relationships is warranted in HER2-low tumors.

Two immune/stromal cell clusters were identified based on CD45 expression and lack of keratin expression. Cluster 3 is characterized by high expression of both CD45 and CD68, suggesting this cluster contains macrophages. Further work to interrogate the phenotype of tumor-associated macrophages may provide an opportunity for new therapeutic targeting^49^. Cluster 6 is less clear but also represents an immune population of cells, likely macrophages, based on its expression of CD68. Both Clusters 3 and 6 also express PD-L1, whereas Cluster 3 has a higher expression of Ki67. Notably, cluster 6 represented 20% of all cells analyzed, which was the highest proportion of total cells in the HER2-enriched cohort of breast tumors. Cluster 1 has heterogeneous expression of most proteins and, therefore did not form a distinct population of cells, as they are spread throughout the t-SNE space. This is likely because sufficient phenotype markers were not included in our antibody panel to accurately identify these cells.

To interrogate the relationship between ITH and clinical outcome, we derived GMM and t-SNE scores from the GMM clustering and t-SNE representation of the CyCIF data. The GMM score is based on the distribution of different cell populations, defined by GMM clustering, and provides a heterogeneity score based on cell-type composition, based on a percentage of cells in each cluster that are present within individual tumors. A limitation of the GMM score is that it may not capture the subtle differences within any given population since it is categorical. For example, cells within the same cluster could be heterogeneous in marker expression, but the GMM score would not capture that. Alternatively, the t-SNE score is defined by the overall distribution in high-dimensional marker space (i.e., t-SNE space), so it should recapitulate more subtle differences between single cells. In most cases, the GMM and t-SNE scores were correlated (Fig. 6g), and we found that both GMM and t-SNE scores correlated with worse clinical outcomes in a historical patient population that was treated with chemotherapy largely without HER2 targeted therapy (Table 7). Importantly, other clinical features such as ER and PR status, age, and tumor grade, all extracted from the clinical data, as well as Ki67 expression derived from CyCIF analysis, did not associate with recurrence, and the clinical stage was only associated in the adjusted analyses (Table 8). This work reveals that single-cell imaging techniques have the ability to define ITH and predict clinical outcomes.

In the current study, HER2+ patients were treated prior to the routine use of Trastuzumab (or other HER2-targeted therapy) and received chemotherapy, hormone therapy, Trastuzumab, or a combination of therapies (Table 7). Future studies are warranted for breast cancer patients who receive (neo)adjuvant anti-HER2 therapy to determine the prognostic and potentially predictive utility of the HER2 ITH and ITH evaluation method developed here. The treatment of HER2+ breast cancer is rapidly evolving and should be taken into consideration for future studies. In addition to anti-HER2 agents, new treatments for HER2+ disease have been tested in the clinic such as Trastuzumab deruxtecan (T-DXd), a HER2 antibody-drug conjugate (ADCs), which is composed of an anti-HER2 antibody, a cleavable tetrapeptide-based linker, and a topoisomerase I inhibitor payload, and have led to remarkable responses in previously treated HER2+ metastatic cancer. In addition, recent data from the Phase 3 DESTINY Breast04 study of patients with HER2-low metastatic breast cancer, T-DXd resulted in significantly longer progression-free and overall survival than the physician’s choice of chemotherapy^50^. Interestingly, T-DXd has recently been shown to work in clinically defined HER2 1–2+^51^ as well as 0^52^ by IHC. The new concept of HER2-low expression level has not yet been defined by ASCO/CAP guidelines, although these patients have been shown to benefit from ADCs^53^. Here, we have described a rigorous approach for assessing ITH, which is likely to be valuable for HER2-low or heterogenous tumors and will need to be tested in these patient cohorts. Single-cell multiplexed tissue imaging may provide an opportunity to interrogate heterogeneity with greater depth in relation to multiple markers and topographic representations and may potentially offer a new approach to assess the duration of clinical benefit in response to HER-targeted therapies.

In the clinical setting, ER, PR, HER2 IHC, and/or FISH are routinely performed on breast tumor samples to inform therapeutic options for the patient. However, even after a tumor is characterized based on the expression of ER, PR, and HER2, clinical studies reveal that response to therapy can vary, in part due to ITH^30^. Our work here indicates that single-cell, multiplexed IF imaging may be a reliable approach to elucidate both HER2 and tumor ITH in research settings and provides a basis for testing multiplexed platforms for assessing ITH in breast tumors in clinical settings. However, additional studies are warranted. A limitation of this study is that we used TMAs instead of whole tissue sections to evaluate ITH, and it is increasingly apparent that whole slide imaging provides a more complete assessment of tumor features, with spatially correlated features resulting in a reduction in effective sample size^40^. However, this analysis of a large number of patients (including 567 HER2+ invasive breast cancer samples from 189 patients with triplicate sampling from each patient) is useful for providing initial insights into the workflows and approaches that can be used to study larger cohorts of whole slide images, as the technical capacity to do so becomes available^54^. Additional analysis on surgical specimens is warranted to investigate ITH at a whole tissue level; however, in the clinical setting, many tumors are sampled by core biopsies that often render limited material, and the statistical approaches needed to account for these small samples require further development. In addition, further work to understand the context of immune and stromal cells, including endothelial cells, fibroblasts, lymphocytes, and innate immune cells, may lend additional information on the complexity of the TME and response to therapy, and these efforts will be facilitated by the use of methodologies that permit deep phenotyping of cellular transcriptomes using emerging single-cell spatial transcriptomic methods.

## Methods

### Specimens, patients, and ethics

#### BC03 TMA

Commercial tissue samples were obtained from Reveal Biosciences (BC03), which includes 16 breast cancer tissues in duplicate with a paired normal tissue. Grading, TNM staging data, AR, ER, PR, HER2, p53, and Ki67 IHC data are available from the vendor.

#### DFCI/BWH TMAs

Breast cancer microarrays were constructed with tissues obtained from untreated, de-identified patients who provided written informed consent under Dana Farber Cancer Institute IRB protocol 93-085. All tissues are from archival excisions or mastectomies, not core biopsies. All tissues are pretreatment (no prior chemotherapy) and were collected between 1998-2005. Archival formalin-fixed, paraffin-embedded breast cancers were collected, and the best blocks and best areas for coring were identified and selected by a breast pathologist (D.D.). Each tumor sample was represented by three tissue microarray cores that, when possible, were taken from different areas of the same tumor. Results of immunohistochemical studies for estrogen (ER) and progesterone receptor (PR) and HER2 and FISH assay results for HER2 were extracted from pathology reports. TMA construction was carried out in the Dana Farber/Harvard Cancer Center Tissue Microarray Core Facility. Three 0.6 mm cores were taken from marked areas and placed into a recipient block using a manual arrayer (Beecher Instruments). Formalin-fixed, paraffin-embedded (FFPE) tissue was sectioned at 5 mm.

#### Ethics

The study was conducted in accordance with ethical principles founded in the Declaration of Helsinki. All analysis was approved by the institutional review boards of Dana-Farber Cancer Institute and Harvard Medical School.

### Reagents and antibodies

To determine the optimal antibody candidate for each biomarker in CyCIF, we compared multiple fluorophore-conjugated antibodies as shown in Tables 1 and 2. Each research (CyCIF) antibody was compared to a single antibody commonly used in clinical practice as a reference.

### Data analyses

Analyses on CyCIF were performed at the level of pixels, cells and tissue cores. In addition, inter-assay analyses were performed comparing: (1) CyCIF *vs*. IHC, the latter assessed both by digital pathology and by two independent pathologists; and (2) CyCIF vs. FISH for HER2. Following validation of these antibodies, the expression of ER, PR, HER2, AR, PD-L1, p53 and Ki67 were used to better understand ITH in breast cancer.

### Single-cell analysis breast cancer cores

For single-cell analysis, a total of 589,343 cells from 278 breast carcinomas were included. In the DFCI TMAs a total of 512,699 cells were analyzed as indicated: HER2+201,601; HR + 94,237; and TNBC 216,861 (Table 6).

### Tissue-based cyclic immunofluorescence

CyCIF (https://www.cycif.org/) was performed as described previously^37^ and used by our group^37,55,56^. Briefly, 4–5 µm FFPE unstained slides were baked (30 mins at 60 °C) and antigen retrieval was performed using Leica BOND RX with ER1 solution (Leica Biosystems #AR9961). A pre-staining cycle is subsequently performed and is constituted by blocking of sample with secondary antibodies so that auto-fluorescence and non-specific antibody binding can be reduced. All staining steps were done at 4 °C overnight. Staining is followed by bleaching with 25 mM NaOH with 4.5% H_2_O_2_ for 45 mins with light exposure. Each successive CyCIF cycle included immunostaining the specimen with the testing antibodies, followed by nuclear staining with a DNA dye, four-channel imaging and fluorophore bleaching. When all cycles are completed, the slide is stained with H&E to allow conventional histopathology review. Individual images are then stitched together into high-dimensional representation for further segmentation and analyses. The RareCyte CyteFinder (RareCyte, Seattle, WA) was used for image capturing. Ashlar (https://github.com/labsyspharm/ashlar) was used to stitch or merge images in each round of CyCIF. This combined image is then viewable using Omero (https://www.openmicroscopy.org/omero/) due to the computational size of the combined image. Single-cell segmentation of the stitched image used the watershed algorithm based on nuclear staining of Hoechst 33342 to generate a nuclear mask image, which defines the single-cell regions extended by 3 pixels to define a cell boundary^35^. Segmentation is based on nuclear stains; however, the cytoplasmic & membrane signals are also captured, relevant for cytoplasmic staining such as HER2, via expanding nuclear masks. The data presented here demonstrate that HER2 positivity from CyCIF is highly correlated with pathologist’s scores indicating this method of segmentation and quantification are representative. Within the single-cell ROIs, gating a ‘positive’ or ‘negative’ status for each marker is conducted based on the local minimum implemented in a custom ImageJ/Matlab script.

### Immunohistochemistry

All IHC was performed in the Brigham and Women’s clinical pathology (CLIA) laboratory. For IHC analyses, 4–5 µm sections were made from FFPE blocks. Unstained slides were deparaffinized and subjected to antigen retrieval using and immunostaining was subsequentially performed with the tested clones (Table 1). All staining procedures were performed according to the manufacturers’ instructions in the presence of appropriate controls. Two pathologists evaluated the IHC expression of each given clone, according to the parameters recommended by the latest protocol from the College of American Pathologist^7^. In addition, IHC was also assessed by digital pathology (Aperio ImageScope by Leica Biosystems Inc.)

### Calculation of Gaussian Mixture Model (GMM) score

All clusters were used to generate the GMM score, which was calculated by the distance matrix from cluster composition of individual patients, and how much deviation from the whole cohort. The formula is:

1 $$\begin{array}{ll}{GMM}\,{score}=1-{distance}\left(\right.{Cohort}[{cluster}\,{composition}],\\{Patient}[{Cluster}\,{composition}]\left.\right)\end{array}$$

As an example:

**Table:** 

	Whole cohort:	Patient 1	Patient 2	Patient 3
Cluster 1:	0.25	0.3	0.1	0.2
Cluster 2:	0.25	0.2	0.3	0.2
Cluster 3:	0.25	0.2	0	0.3
Cluster 4:	0.25	0.3	0.6	0.3

In this case, patients 1 & 3 are with GMM score 0.9, while patient 2 is 0.54. The lower the score, the more heterogeneous.

### Calculation of t-distributed stochastic neighbor embedding (t-SNE) score

All clusters were used to generate the t-SNE score, which was done by Cyt package as described^37^. After generating the tSNE1/tSNE2 values for each single cells, the t-SNE score for each TMA cores was calculated used the formula below:

2 $${tSNE}\,{score}=\sqrt{\sum{\left(\right.{tSNE}1-{mean}({{tSNE}1}_{{all}\,{cells}})}^{2}+\sum {\left(\right.{tSNE}2-{mean}({{tSNE}2}_{{all}\,{cells}})}^{2}}$$

### Association of clinical data

Some levels of clinical stage and tumor grade were combined due to numbers of patients in some groups. To test the association between GMM/t-SNE score and other features, the following methods were used:

1. For ER, PR and tumor grade, Wilcoxon rank-test was used due to data having two categories.
2. For clinical stage the Kruskal-Wallis test was used.
3. For age and CyCIF tumor Ki67 analysis the Spearman correlation test was used.

Cox proportional hazard model was used to fit two models, one with GMM score and clinical features; and the other with t-SNE score and clinical features. The hazard ratio and *p* value are shown.

### Supplementary information

Suppl. Figures

Related Manuscript File

Supplementary Table 1

## Data Availability

The data that support the findings of this study are available upon reasonable request from the corresponding author (J.L.G.). All CyCIF images are available at https://www.tissue-atlas.org/atlas-datasets/guerriero-lin-santagata-2023.

## Code availability

All code used in the analysis are available at https://github.com/labsyspharm/npjbcancer2023.
