# Supplementary material for: Qualification of a multiplexed tissue imaging assay and detection of novel patterns of HER2 heterogeneity in breast cancer
Source: NPJ Breast Cancer. 2024 Jan 2;10:2. doi: 10.1038/s41523-023-00605-3 (PMC10761880; doi:10.1038/s41523-023-00605-3)
Supplement: Supplementary file 1 — Suppl. Figures [file 41523_2023_605_MOESM1_ESM.pdf]

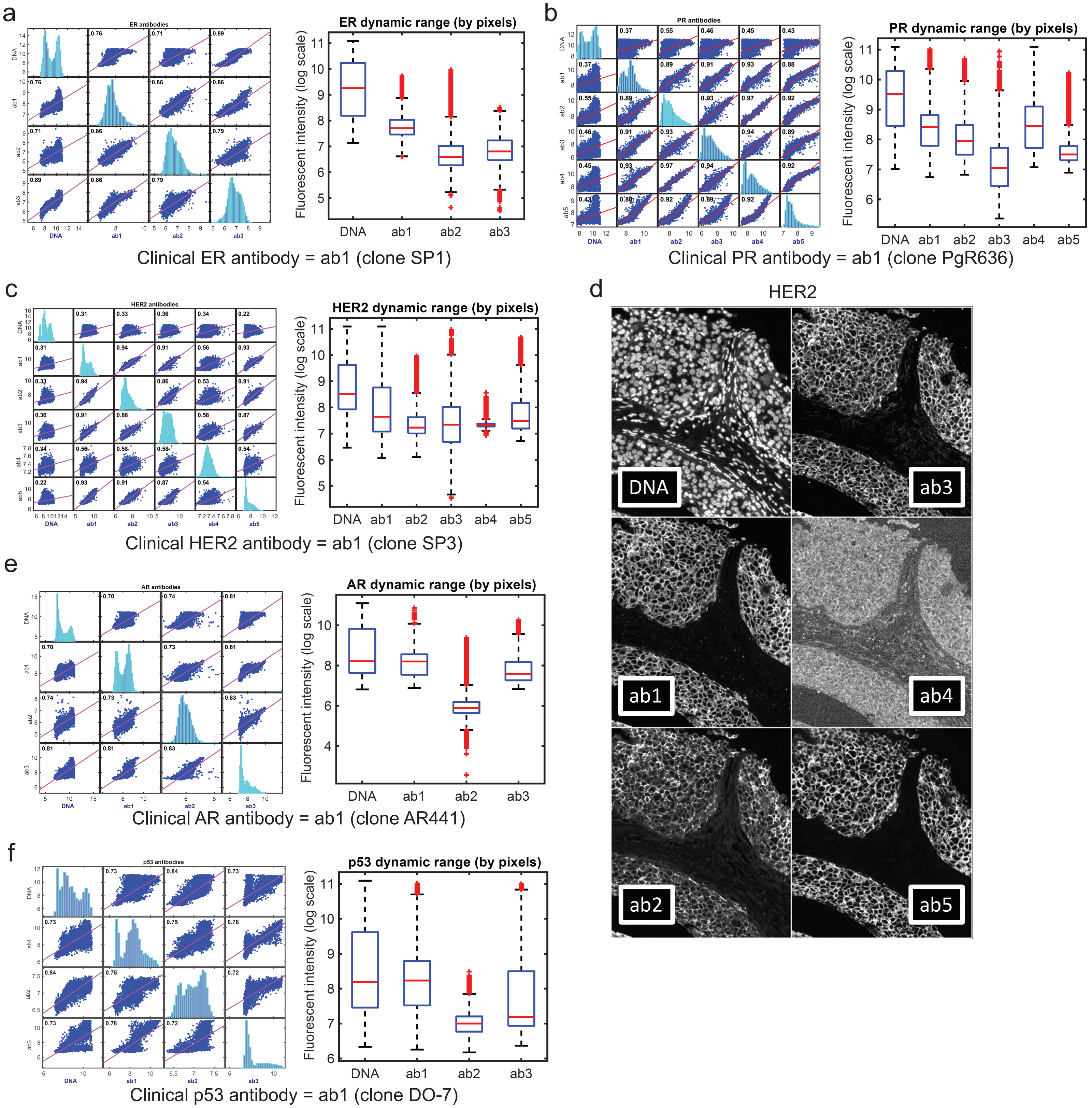

Supplementary Figure 1. Pixel-level analysis of clinical and CyCIF antibodies against ER, PR, HER2, AR and p53 in the BC03 TMA. Pixel-to-pixel comparison was performed between multiple CyCIF fluorescently conjugated antibodies against a single antibody commonly used in clinical practice as a reference. Ab1 refers to the clinical antibody, which was used in the first cycle of CyCIF in unconjugated form, followed by a secondary antibody. CyCIF antibodies were used in subsequent cycles. A region of interest (ROI) was selected, and single-pixel data was extracted, and cross-image comparisons are shown for (A) ER, (B) PR, (C) HER2, (D) HER2 CyCIF images (E) AR and (F) AR. Each dot represents one pixel. Pink lines represent the linear regression and the r scores using Pearson correlation are labeled in each inset. DNA/Hoechst staining is used as the negative control. Right graph, to test the sensitivity of each fluorescent antibody, the pixel data is extracted as described above and are plotted using default BOXPLOT function in MATLAB. The median values, 75%/25% percentiles and 95%/5% percentiles are indicated with the red line, box margins and whiskers. The sensitives of each antibody are estimated from the range between 75% and 25% or between 95% and 5%.

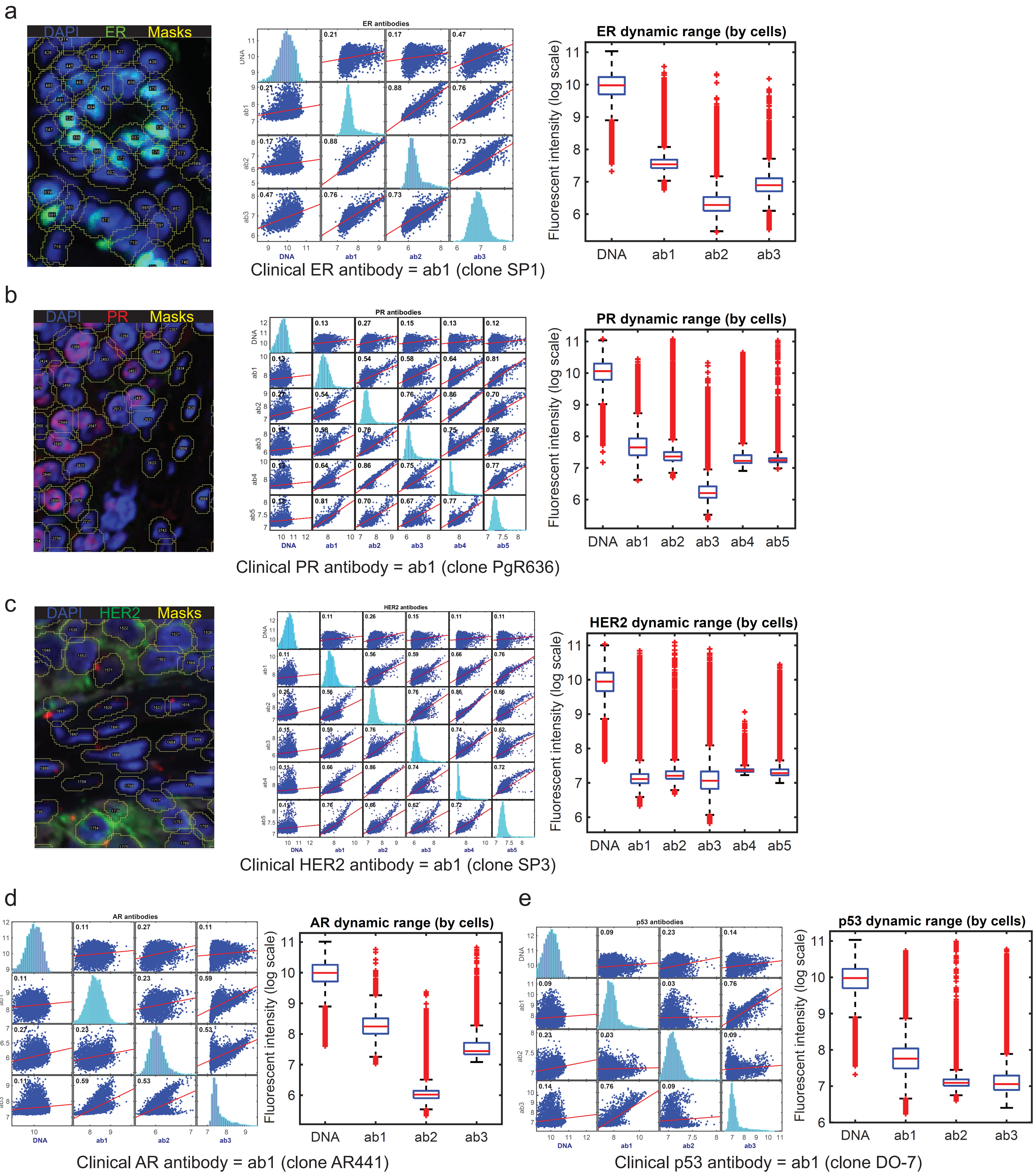

Supplementary Figure 2. Single-cell-to-cell comparison of clinical and CyCIF antibodies against ER, PR, HER2, AR and p53 using the BC03 TMA. Cell-to-Cell comparison was performed between multiple CyCIF fluorescently conjugated antibodies against a single antibody commonly used in clinical practice as a reference. Ab1 refers to the clinical antibody, which was used in the first cycle of CyCIF in unconjugated form, followed by a secondary antibody. Single-cell data were extracted as described in the Methods section. (A-C) Representative images of the clinical antibodies and the segmentation masks used to obtain single-cell data are shown in the graph on the left for (A) ER, (B) PR and (C) HER2. Middle graph shows cell-to-cell comparison of intensity of each cell in log scale. The pink lines represent the linear regression and the r scores using Pearson correlation are shown in each graph. Graph on the right shows the dynamic range of cell positive across each antibody. The median values, 75/25 percentiles and 95/5 percentiles are indicated with the red line, box margins and whiskers. Outlier cells shown in red, above and below boxes. (D-E) Same as middle and right graphs above, for (D) AR and (E) p53.

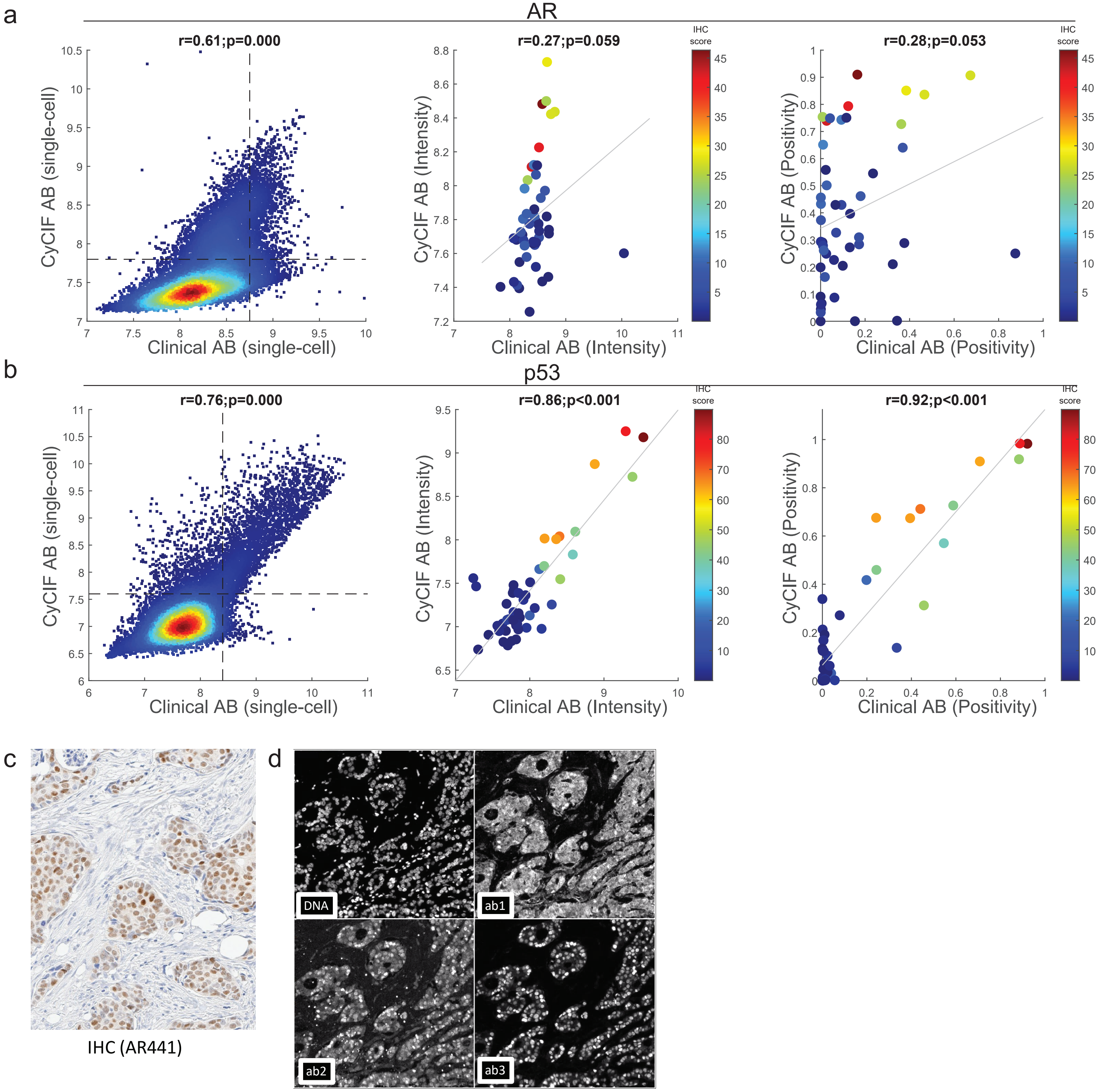

Supplementary Figure 3. Core-to-core comparison of clinical and CyCIF antibodies against AR and p53. To qualify breast cancer related antibodies AR and p53, the BC03 TMA, representing 16 breast tumors in duplicate was used. CyCIF was performed using the qualified CyCIF antibody against a single antibody commonly used in clinical practice as a reference. (A-B) The left graph depicts a single-cell dot-plot between the clinical clone on the x-axis and validated CyCIF antibody on the y-axis. Each dot represents single-cell fluorescent intensity values from the 2 antibodies. Dashed lines indicate the gating strategy. The middle graph shows the corresponding mean log intensity of the core-to-core analysis of the clinical and CyCIF antibodies. The single-cell data were collected for individual TMA cores, with a binary gate applied to obtain the positive signal of each core (range from 0-1). The X- & Y- axis represent the positive score calculated from either clinical or CyCIF antibodies, respectively. The right graph shows positivity scores (number of positive cells over total cells) for the clinical and CyCIF antibodies by TMA case. (C) Representative image of the clinical AR antibody by IHC. (D) Representative images of the AR clinical antibody (ab1) compared to two CyCIF antibodies shown using the CyCIF protocol. Note that the clinical AR antibody underperforms in the CyCIF assay.

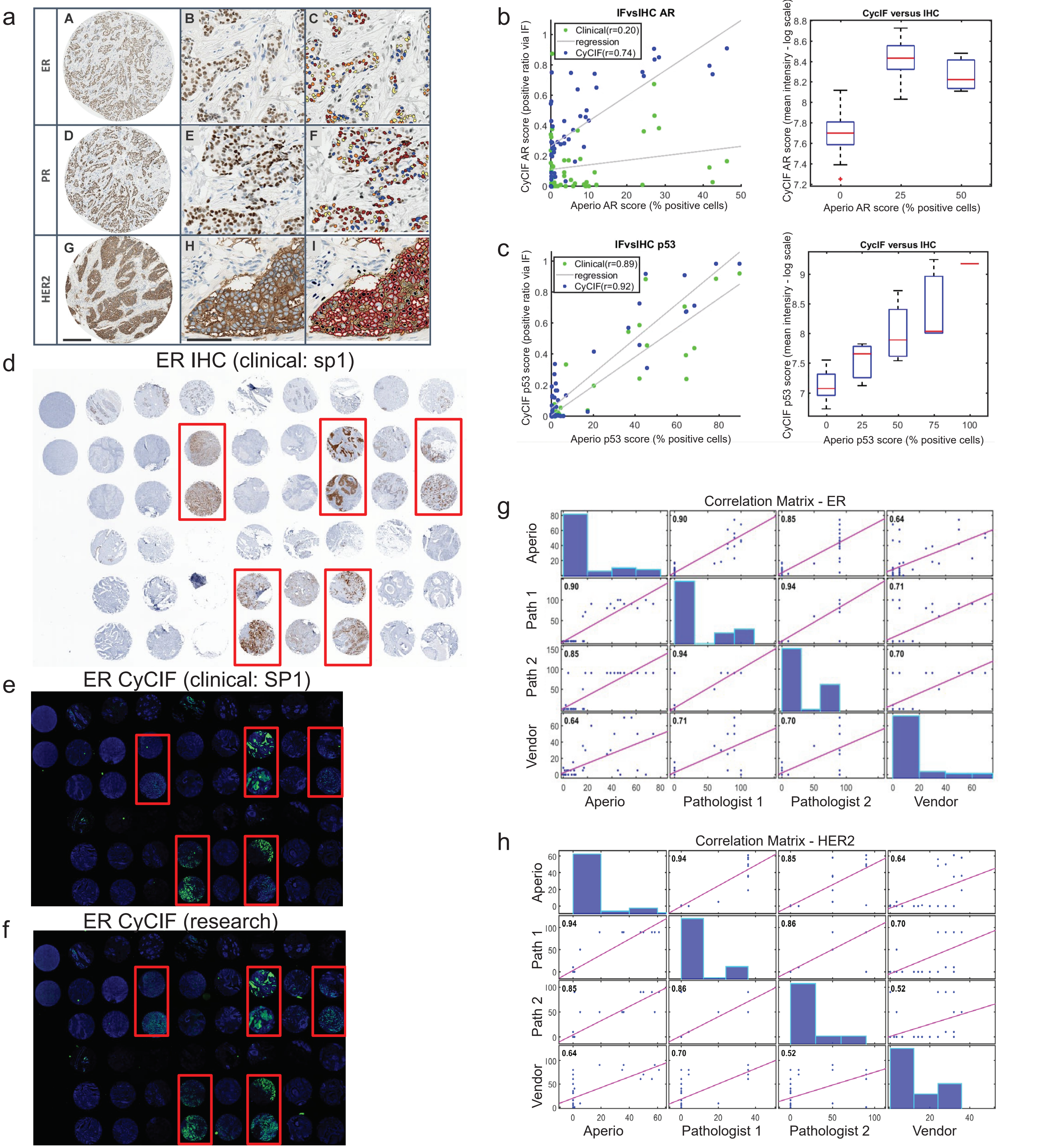

Supplementary Figure 4. IHC analysis and Aperio Scoring of BC03 TMA. The BC03 TMA was stained with clinical and CyCIF antibodies using the CyCIF protocol and serial sections were stained with clinical antibodies using standard IHC in the clinical laboratory. (A) Representative images of ER/PR/HER2 staining by IHC. (B-C) Cross-assay comparison of the clinical and CyCIF antibodies analyzed by CyCIF compared to the clinical antibody analyzed by IHC using Aperio software for AR (B) and p53 (C). Left, Dot-plot represents two different scores obtained from CyCIF and one obtained from IHC-Aperio. CyCIF of clinical (green dots) and CyCIF (blue dots) antibodies were used on the same section, while IHC was done on a different section from the same TMA block. Each dot represents a single core from BC03 TMA. CyCIF scores are plotted on y-axis, IHC scores on x-axis. Right, quantitative assessment of AR and p53 IHC versus CyCIF staining. IHC scores by Aperio were used to stratify different TMA cores/cases and the mean intensities of CyCIF antibody staining from each TMA core are shown using boxplot analysis. CyCIF antibodies: AR (ab194195) and p53 (ab224942). (D-F) Images of IHC using the clinical ER antibody (sp1; D), CyCIF using the clinical ER antibody (sp1; E) and CyCIF using the CyCIF antibody (F). (G-H) Correlation between ER (G) & HER2 (H) from clinical antibodies scored using Aperio software, two independent pathologists (path1 and path2) and the scores provided by TMA commercial vendors (no information is available regarding antibodies used by the vendor). The pink lines represent the linear regression and the r scores using Spearman correlation are labeled in each inset.

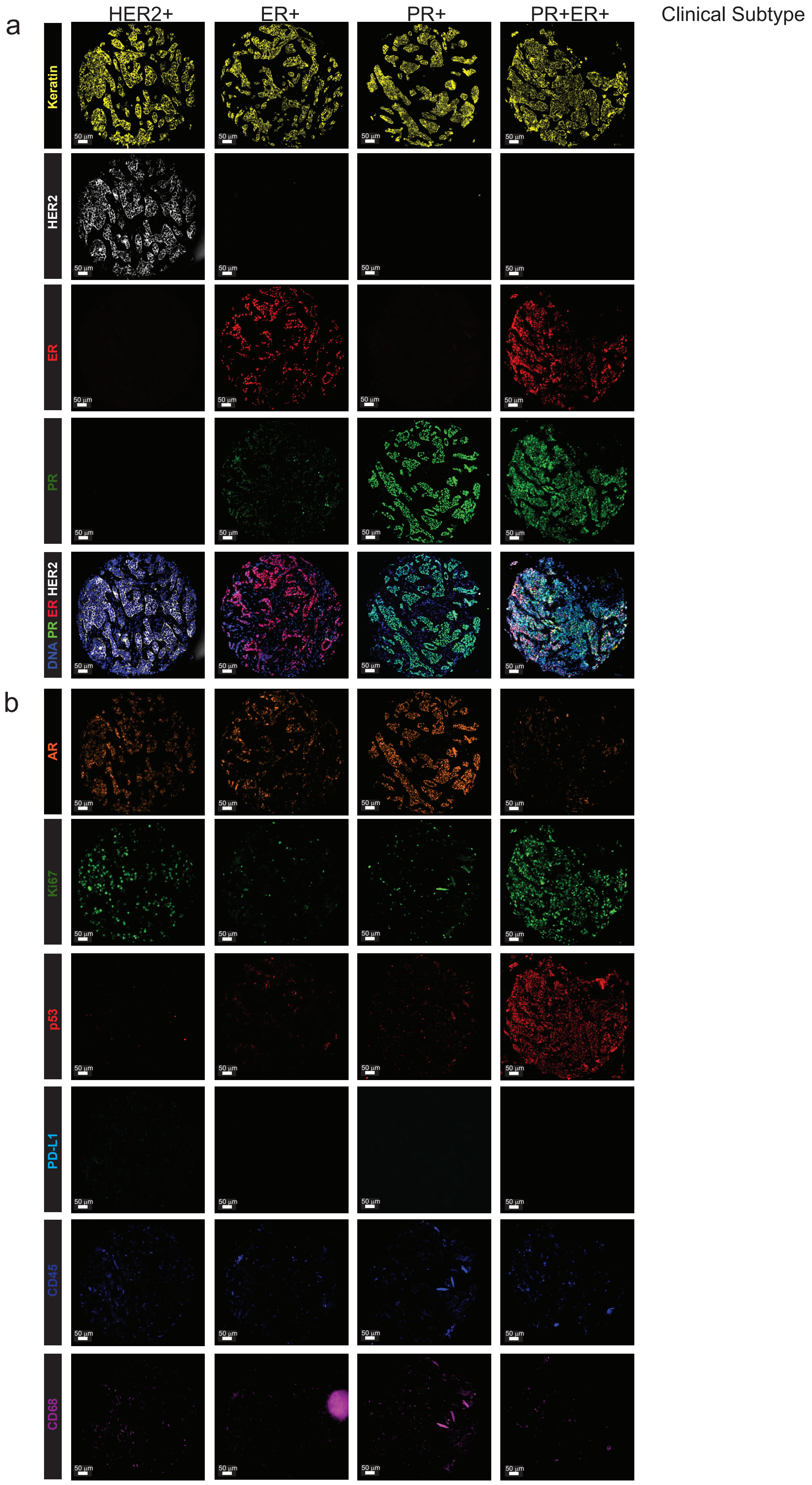

Supplementary Figure 5. CyCIF images of representative tissues. Tissues with different clinical annotations of HER2+, ER+ and PR+ were selected to show corresponding staining of individual CyCIF antibodies.

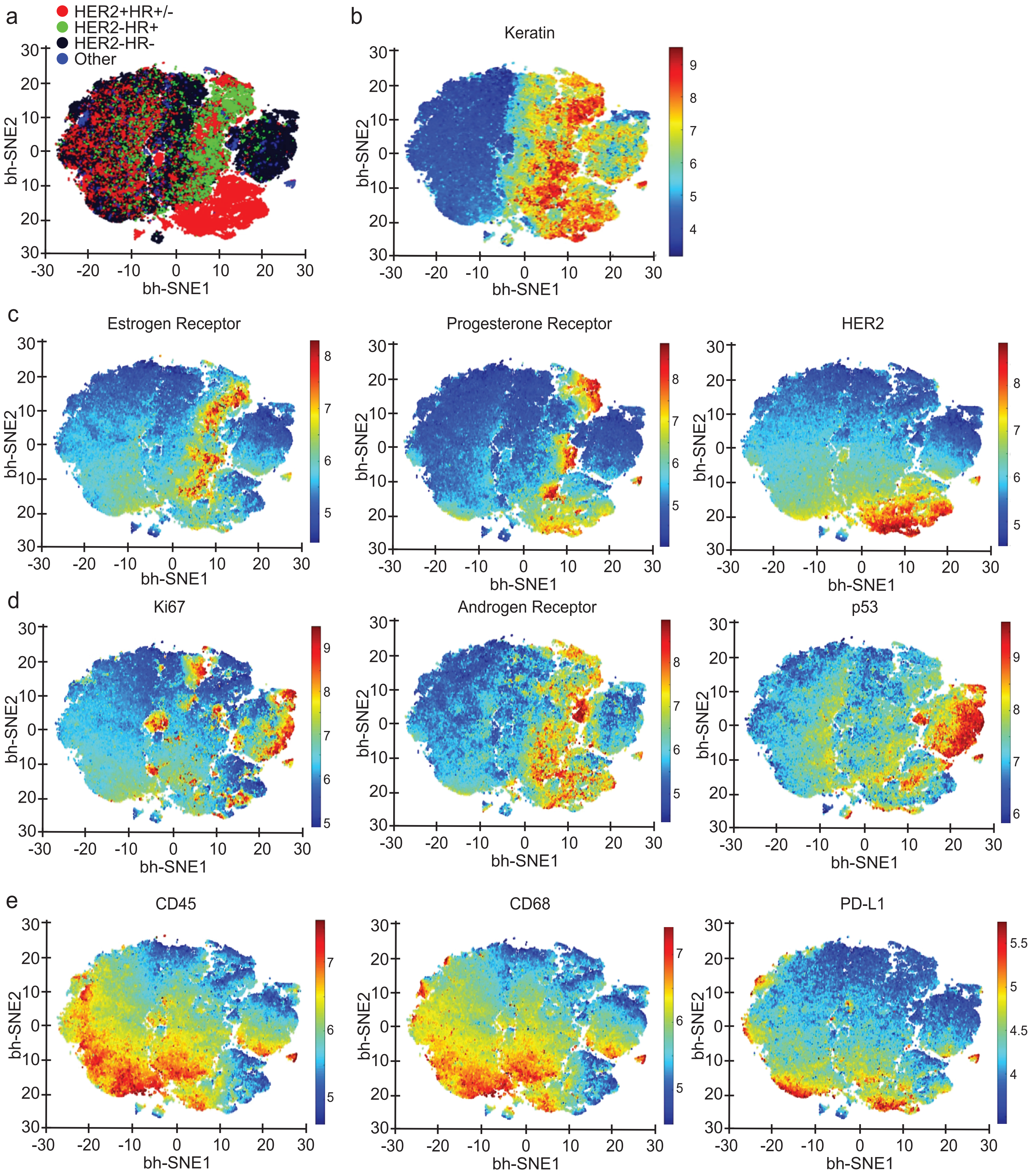

Supplementary Figure 6. Overview of single-cell data from TMAs 240, 226, 227. A total of 834 breast tumors, representing 278 patients in triplicate, were analyzed at the single-cell level using CyCIF, pooling all cells from each of the triplicate cores. (A) All cells are shown defined by t-SNE are color coded by clinical annotation and cluster by HR/HER2 status. (B-E) Markers of interest are shown in t-SNEs.
