# Supplementary material for: Qualification of a multiplexed tissue imaging assay and detection of novel patterns of HER2 heterogeneity in breast cancer
Source: NPJ Breast Cancer. 2024 Jan 2;10:2. doi: 10.1038/s41523-023-00605-3 (PMC10761880; doi:10.1038/s41523-023-00605-3)
Supplement: Supplementary file 2 — Related Manuscript File [file 41523_2023_605_MOESM2_ESM.pdf]

Corresponding author(s): Jennifer L. Guerriero, PhD

Last updated by author(s): Nov 9, 2023

Reporting Summary

Nature Portfolio wishes to improve the reproducibility of the work that we publish. This form provides structure for consistency and transparency in reporting. For further information on Nature Portfolio policies, see our Editorial Policies and the Editorial Policy Checklist.

Statistics

For all statistical analyses, confirm that the following items are present in the figure legend, table legend, main text, or Methods section.

n/a	Confirmed	
☐	☒	The exact sample size (*n*) for each experimental group/condition, given as a discrete number and unit of measurement
☐	☒	A statement on whether measurements were taken from distinct samples or whether the same sample was measured repeatedly
☐	☒	The statistical test(s) used AND whether they are one- or two-sided<br>*Only common tests should be described solely by name; describe more complex techniques in the Methods section.*
☐	☒	A description of all covariates tested
☐	☒	A description of any assumptions or corrections, such as tests of normality and adjustment for multiple comparisons
☐	☒	A full description of the statistical parameters including central tendency (e.g. means) or other basic estimates (e.g. regression coefficient) AND variation (e.g. standard deviation) or associated estimates of uncertainty (e.g. confidence intervals)
☐	☒	For null hypothesis testing, the test statistic (e.g. *F*, *t*, *r*) with confidence intervals, effect sizes, degrees of freedom and *P* value noted<br>*Give P values as exact values whenever suitable.*
☐	☒	For Bayesian analysis, information on the choice of priors and Markov chain Monte Carlo settings
☐	☒	For hierarchical and complex designs, identification of the appropriate level for tests and full reporting of outcomes
☐	☒	Estimates of effect sizes (e.g. Cohen's *d*, Pearson's *r*), indicating how they were calculated

Our web collection on statistics for biologists contains articles on many of the points above.

Software and code

Policy information about availability of computer code

Data collection	All code used in the analysis are available at https://github.com/labsyspharm/npjbcancer2023
Data analysis	All code used in the analysis are available at https://github.com/labsyspharm/npjbcancer2023

For manuscripts utilizing custom algorithms or software that are central to the research but not yet described in published literature, software must be made available to editors and reviewers. We strongly encourage code deposition in a community repository (e.g. GitHub). See the Nature Portfolio guidelines for submitting code & software for further information.

Data

Policy information about availability of data

All manuscripts must include a data availability statement. This statement should provide the following information, where applicable:

- Accession codes, unique identifiers, or web links for publicly available datasets
- A description of any restrictions on data availability
- For clinical datasets or third party data, please ensure that the statement adheres to our policy

The data that support the findings of this study are available upon reasonable request from the corresponding author (J.L.G.). All CyCIF images are available at https://www.tissue-atlas.org/atlas-datasets/guerriero-lin-santagata-2023

Human research participants

Policy information about studies involving human research participants and Sex and Gender in Research.

Reporting on sex and gender	Female breast cancer patients were used in this collection cohort.
Population characteristics	n/a
Recruitment	Breast cancer microarrays were constructed with tissues obtained from untreated, de-identified patients who provided written informed consent under Dana Farber Cancer Institute IRB protocol 93-085. Archival formalin-fixed, paraffin-embedded breast cancers were collected and best blocks and best areas for coring were identified and selected by a breast pathologist (D.D.). Each tumor sample was represented by three tissue microarray cores that, when possible, were taken from different areas of the same tumor.
Ethics oversight	Dana-Farber Cancer Institute (93-085).

Note that full information on the approval of the study protocol must also be provided in the manuscript.

Field-specific reporting

Please select the one below that is the best fit for your research. If you are not sure, read the appropriate sections before making your selection.

☒ Life sciences ☐ Behavioural & social sciences ☐ Ecological, evolutionary & environmental sciences

For a reference copy of the document with all sections, see nature.com/documents/nr-reporting-summary-flat.pdf

Life sciences study design

All studies must disclose on these points even when the disclosure is negative.

Sample size	Sample size was based on tissue available from cores made on 93-085. 866 tissue cores (representing 294 patients in triplicate).
Data exclusions	Tissue microarray cores were excluded if there were less than 500 cells available for analysis.
Replication	The BC03 commercial tissue array (48 tissue cores (representing 24 tumors in duplicate)) was used for qualifying CyCIF antibodies and then DFCI tissue collection cohort samples from 93-085 were used as validation (866 tissue cores (representing 294 patients in triplicate)).
Randomization	N/A
Blinding	N/A

Reporting for specific materials, systems and methods

We require information from authors about some types of materials, experimental systems and methods used in many studies. Here, indicate whether each material, system or method listed is relevant to your study. If you are not sure if a list item applies to your research, read the appropriate section before selecting a response.

Materials & experimental systems

n/a	Involved in the study	
☐	☒	Antibodies
☒	☐	Eukaryotic cell lines
☒	☐	Palaeontology and archaeology
☒	☐	Animals and other organisms
☐	☒	Clinical data
☒	☐	Dual use research of concern

Methods

n/a	Involved in the study	
☒	☐	ChIP-seq
☒	☐	Flow cytometry
☒	☐	MRI-based neuroimaging

Antibodies

Antibodies used	Antibodies are detailed in Table 1.
Validation	The validation is part of the main text.

Clinical data

Policy information about clinical studies

All manuscripts should comply with the ICMJE guidelines for publication of clinical research and a completed CONSORT checklist must be included with all submissions.

Clinical trial registration	n/a
Study protocol	n/a
Data collection	93-085
Outcomes	n/a
